# Comparing the Effects of Dairy and Soybean on Bone Health in Women: A Food- and Component-Level Network Meta-Analysis

**DOI:** 10.3390/nu17172833

**Published:** 2025-08-30

**Authors:** Li You, Langrun Wang, Shiwen Zhou, Yiran Guan, Yan Liu, Ruixin Zhu, Huiyu Chen, Jie Guo, Keji Li, Xingyu Bao, Haotian Feng, Ignatius M. Y. Szeto, Jian He, Ran Wang, Jingjing He

**Affiliations:** 1 Key Laboratory of Precision Nutrition and Food Quality, Department of Nutrition and Health, China Agricultural University, Beijing 100193, China; youli@ccibe.edu.cn (L.Y.); b20243311365@cau.edu.cn (L.W.); zhoushiweeeeen@163.com (S.Z.); sy20233313730@cau.edu.cn (Y.G.); zhuruixin07@126.com (R.Z.); huiyuchen611@gmail.com (H.C.); guojie@cau.edu.cn (J.G.); 2 College of Physical Education and Health, Chongqing College of International Business and Economics, Chongqing 401520, China; 3 National Center of Technology Innovation for Dairy, Hohhot 010100, China; liuyan37@yili.com (Y.L.); baoxingyu@yili.com (X.B.); fenghaotian@yili.com (H.F.); szeto@yili.com (I.M.Y.S.); hejian@yili.com (J.H.); 4 Inner Mongolia Dairy Technology Research Institute Co., Ltd., Hohhot 010100, China; 5 Inner Mongolia Yili Industrial Group Co., Ltd., Hohhot 010100, China; 6 Department of Nutrition and Food Hygiene, School of Public Health, Peking University, Beijing 100191, China; kejili@bjmu.edu.cn; 7 Research Center for Probiotics, China Agricultural University, Beijing 100193, China

**Keywords:** bone health, dairy, soybean, network meta-analysis

## Abstract

**Background/Objectives**: Dairy and soybean are important potential dietary sources of bone health. However, their comparative effectiveness and the role of specific components remain unclear. In this network meta-analysis (NMA), we aimed to compare the effects of various dairy and soy products (food level) and their key bioactive components (component level) on bone health in healthy women. **Methods**: We systematically searched PubMed, Embase, Cochrane Library, and Web of Science (up to 28 February 2025) for randomized controlled trials. A frequentist random-effects NMA was used to compare interventions for lumbar spine (LS) and total body (TB) bone mineral density (BMD) and bone turnover markers [osteocalcin (OC), deoxypyridinoline (DPD)]. Mean differences (MDs) and 95% confidence intervals were pooled. Interventions were ranked using the surface under the cumulative ranking curve (SUCRA). **Results**: Sixty RCTs involving 6284 participants (mean age: 54.2 years) were included. At the food level, no dairy or soy interventions significantly improved outcomes versus control, although milk + yogurt ranked numerically highest based on SUCRA values. At the bioactive-component level, the combination of casein + whey protein (MD 0.04 g/cm^2^, 95% CI 0.01–0.06) and soybean protein (MD: 0.03 g/cm^2^, 95% CI: 0.01–0.05) significantly increased TB BMD. Whey protein alone (SUCRA 74.4% for LS BMD) and casein + whey protein (SUCRA 86.3% for TB BMD and 75.9% for DPD) were among the highest-ranked interventions for bone health. **Conclusions**: The combination of milk and yogurt may be relatively promising among dairy products for bone health. Whey protein appears to be a key bioactive component beneficial for women’s bone health.

## 1. Introduction

Bone is a dynamic tissue that undergoes continuous remodeling throughout life, involving formation and resorption [1,2]. While bone formation predominates during growth, bone resorption increases after age 40, particularly in women experiencing postmenopausal estrogen decline, elevating the risk of osteoporosis and fractures due to reduced bone mineral density (BMD) and strength [3,4]. Osteoporosis, characterized by low BMD and deteriorated bone microarchitecture, disproportionately affects women, highlighting the critical need for effective strategies to maintain bone health [5,6]. Beyond estrogen deficiency, obesity and related chronic low-grade inflammation are recognized as critical modifiable risk factors for osteoporosis [7]. These conditions disrupt the bone marrow microenvironment—altering stem cell differentiation, impairing osteogenesis while promoting adipogenesis—thereby compromising bone remodeling balance [8]. Furthermore, specific dietary patterns (e.g., “milk/dairy” patterns) have been associated with a reduced risk of low BMD and fracture [9,10]. Consequently, effective nutritional strategies for bone health should not only deliver key nutrients but also account for their potential to modulate systemic inflammatory status and be embedded within beneficial dietary patterns.

Dairy products (e.g., milk, yogurt, powder) and soybeans are widely consumed and recognized as potential dietary supports for bone health [11,12,13,14]. Dairy is rich in protein, calcium, and vitamin D [15], while soy provides high-quality plant protein and isoflavones with estrogen-like properties that may influence bone cell activity [16,17,18]. Critically, processing milk or soybeans into different forms (e.g., liquid milk vs. milk powder, whole soybeans vs. soymilk) alters the content and bioavailability of key components like whey proteins [19] and isoflavones [20]. Consequently, the effects of various dairy and soy products on bone health may differ. Furthermore, consumer preferences vary; individuals following vegan/vegetarian diets or with lactose intolerance/milk allergies may avoid dairy [21,22], creating a need to understand the relative efficacy of soy alternatives. However, the current evidence has dual limitations: it neither establishes the comparative superiority between dairy and soy products overall nor provides a systematic ranking of the efficacy of different product forms within each category (e.g., milk vs. yogurt vs. milk powder; soybeans vs. soymilk vs. tofu).

Beyond variations in product forms, elucidating the mechanistic roles of their core bioactive components is critical for developing targeted nutritional interventions. At the bioactive-component level, while guidelines recommend the consumption of protein, calcium, and vitamin D to support bone health [23], evidence regarding the efficacy of calcium or vitamin D supplementation alone remains inconsistent. Several systematic reviews and meta-analyses have demonstrated that vitamin D and calcium supplementation do not have beneficial effects on bone health [24,25], including in postmenopausal women [26]. In contrast, proteins have exhibited benefits for bone health, although the research results are heterogeneous [27,28,29]. Notably, milk protein and soy protein differ in their amino acid composition [30], and recent comparative studies of animal versus plant-based proteins have produced conflicting results, often without differentiating between specific protein types or focusing on women specifically [31]. The distinct effects of soy isoflavones also remain to be fully elucidated. Furthermore, although both protein and isoflavones are recognized as key bioactive components, their relative contributions—particularly when comparing specific milk proteins (such as whey and casein) with soy protein and isoflavones—have not been adequately evaluated.

Network meta-analysis (NMA) is an advanced statistical technique uniquely suited to address these gaps. Unlike traditional pairwise meta-analyses, NMA allows for the simultaneous comparison and ranking of multiple interventions by synthesizing both direct (head-to-head trials) and indirect evidence (trials connected through common comparators) within a unified analysis [32,33,34]. This capability is crucial for comprehensively evaluating the diverse range of dairy and soy products and their bioactive components relevant to bone health. This study thus aims to achieve the following outcomes: (1) at the food-class level, compare the effects of various dairy and soy products on bone health; and (2) at the bioactive-component level, evaluate the relative contributions of specific components (milk proteins: casein, whey, and milk basic protein; soy protein; soy isoflavones).

## 2. Materials and Methods

The present study adhered rigorously to the Preferred Reporting Items for Systematic Reviews and Meta-analyses (PRISMA) Statement [35]. It was registered in the Prospective Register of Systematic Reviews (PROSPERO) under the registration number CRD42024560199 (https://www.crd.york.ac.uk/prospero/display_record.php?ID=CRD42024560199), as of 5 July 2024.

### 2.1. Search Strategy

We systematically searched PubMed, Embase, Cochrane Library, and Web of Science (up to 28 February 2025). The keywords and MeSH terms used in the search strategy included dairy products, milk, cultured milk products, butter, buttermilk, cheese, Kefir, Koumiss, yogurt, milk proteins, whey proteins, caseins, lactalbumin, lactoglobulins, lactoferrin, osteopontin, glycine max, soybean proteins, isoflavones, equol, bone density, bone remodeling, osteogenesis, bone resorption, and osteoporosis. The full search strategies used in the above databases are provided in Tables S1–S4.

### 2.2. Inclusion and Exclusion Criteria

Studies were included if they met the following criteria:

(1) Participants were healthy female adults aged ≥18 years, which included those with overweight or obesity but excluded individuals with other diseases known to affect bone metabolism (see exclusion criteria 2 below).
(2) Study design was a randomized controlled trial (RCT).
(3) Intervention groups received single or combined treatments of dairy products (milk, yogurt, milk powder, cheese), milk-derived proteins (milk protein, casein, whey protein, milk basic protein), soybean, soy protein, or soy isoflavones.
(4) Control groups received no treatment, placebo, or any intervention listed in (3).
(5) Data for ≥1 bone health outcome (BMD or bone turnover markers) were reported or calculable.

Primary outcome: BMD of the whole body or any specific site.

Secondary outcomes: Bone turnover markers (osteocalcin [OC], bone-specific alkaline phosphatase [BAP], procollagen type I *N*-terminal propeptide [PINP], deoxypyridinoline [DPD], *C*-terminal telopeptide of type I collagen [CTx], *N*-terminal telopeptide of type I collagen [NTx], etc.) and bone metabolism hormones (parathyroid hormone [PTH], insulin-like growth factor 1 [IGF-1], 25-hydroxyvitamin D [(25(OH)D]).

Exclusion criteria:

(1) Non-English publications, abstracts, letters, conference reports, or duplicate publications.
(2) Studies involving women with any disease that affects bone metabolism, including renal failure, liver disorders, hyperparathyroidism, hyperthyroidism, diabetes mellitus, or cancer.
(3) Interventions confounded by nontarget components (e.g., vitamin D).
(4) Cointerventions (e.g., exercise programs).
(5) No complete data regarding the effect sizes could be extracted from this study, nor could such information be derived through reliable methods.

### 2.3. Data Extraction

EndNote X9.2 was employed for the screening and management of the literature. Following predefined inclusion and exclusion criteria, two researchers (L.Y. and S.W.Z.) independently performed the initial screening using the titles and abstracts, and subsequently reviewed the full texts to decide on the inclusion of the studies. A third researcher (J.J.H.) was consulted in case of disagreement in the selection of studies.

Two researchers (S.W.Z. and L.Y.) independently extracted data as outlined in the Cochrane handbook. The extracted details included study title, authors, year of publication, country, participant description (race/ethnicity, age, and BMI), intervention period, sample size, treatment method for the intervention or the control group, and outcome indicators (mean and standard deviation [SD] of the change from baseline to endpoint in BMD and bone turnover markers). When mean was not reported, we estimate it using the median (quartile) or the median (minimum, maximum) as appropriate [36]. When SD was not directly reported, it was calculated from the standard error or confidence interval (CI) following the Cochrane Handbook for Systematic Reviews of Interventions [37]. If the mean or SD could not be obtained through a reliable method, the data were not included in the meta-analysis. Disagreements were resolved through consensus with the third researcher (J.J.H.).

### 2.4. Risk-of-Bias Assessment

The quality of the included studies was assessed using the Risk of Bias tool (RoB 2) and the Cochrane Risk of Bias Assessment Tool [38]. The evaluation criteria encompassed five components: randomization process, deviations from intended interventions, missing outcome data, measurement of the outcome, and selection of the reported result. Each component was assessed as ‘low risk’, ‘high risk’, or ‘uncertain’ [34,39]. The process of evaluating the quality of the literature was carried out independently by two researchers (L.Y. and L.R.W.), and in case of disagreement, a third researcher (J.J.H.) was added to discuss the matter together until there was unanimity of opinion.

### 2.5. Statistical Analysis

We conducted two independent frequentist random-effects network meta-analyses (NMAs) using Stata 17.0 and R 4.3.1: one at the food-class level (comparing dairy products and soy products) and another at the bioactive-component level (comparing milk-derived protein, soybean protein, and soy isoflavones). Empirical and simulation studies have demonstrated that, in most scenarios, the frequentist and Bayesian NMA methodologies typically yield consistent results [40,41]. We chose the frequentist approach due to its ease of implementation and the availability of a comprehensive set of tools in the Stata software platform. Furthermore, this approach is consistent with the practices adopted in recent high-quality network meta-analyses [42].

Statistical significance was set at *p* < 0.05. Based on the overall characteristics of the included literature, this study’s NMAs focused on the BMD values of LS and TB, as well as the bone turnover markers OC and DPD. For the BMD of other body parts and other bone turnover markers such as CTx and P1NP, NMA was not performed due to a limited number of original studies or intervention comparisons.

All outcome measures in this study were continuous variables; therefore, the mean differences (MDs) and SDs between the intervention group and the control group were utilized as effect sizes for each trial comparison. We employed a design-by-treatment interaction model as the global approach and a loop-specific method alongside a side-splitting model as the local approach to assess inconsistency within the network [43,44,45]. If the *p*-value from the chi-square test in the global inconsistency assessment was <0.05, a consistency model was adopted for the NMA. Conversely, an inconsistency model was utilized, necessitating further investigation into potential sources of inconsistency. For each outcome measure with sufficient data, the surface under the cumulative ranking curve (SUCRA) was used to rank the efficacy of different interventions, with higher values (ranging from 0% to 100%) indicating superior efficacy.

Sources of heterogeneity were explored using subgroup analysis and sensitivity analysis. Subgroup analysis was performed for each outcome that included at least three studies according to BMI values (<24.0 vs. ≥24.0 kg/m^2^). When significant local inconsistencies arise during loop analysis, sensitivity analyses were performed by excluding studies contributing to inconsistent loops. Publication bias was assessed using funnel plots combined with Egger’s tests for outcome measures involving more than 10 comparisons.

## 3. Results

### 3.1. Literature Search and Screening

**Figure 1:**
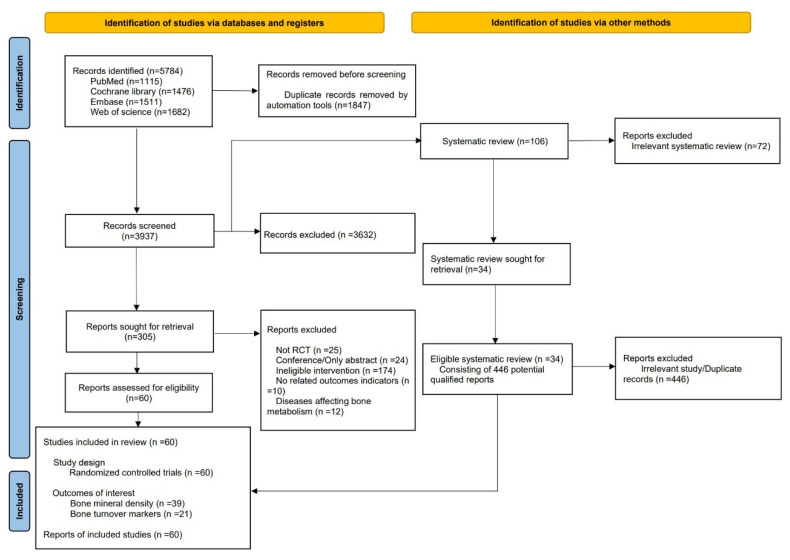
Flow diagram of literature search and screen.

Initial database searches identified 5784 records. After removing duplicates (EndNote X9.2), 3937 records remained. Screening of titles and abstracts excluded 3632 records as irrelevant. Full-text assessment of the remaining 305 records excluded 245 records not meeting the inclusion criteria, resulting in 60 records for final inclusion (Figure 1).

### 3.2. Study Characteristics

As shown in Table 1, the included RCTs (1995–2022) involved 6284 healthy women. The mean age of the participants was 54.2 years, with a range from 19.6 to 74.2 years. The average body mass index (BMI) was 25.1 kg/m^2^, ranging from 20.4 to 32.2 kg/m^2^. The duration of the interventions averaged 11.3 months.

Thirteen intervention groups were analyzed: control (placebo/no treatment), soybean protein, isoflavone, soybean protein + isoflavone, milk basic protein (MBP), casein, whey protein, casein + whey protein, milk, yogurt, milk + yogurt, milk powder, soymilk.

Table S5 summarizes the distribution of included study outcomes. Among the sixty included RCTs, BMD was assessed for the lumbar spine (LS; 32 RCTs); total body (TB; 20 RCTs); femoral neck (FN; 24 RCTs); total hip (TH; 16 RCTs); trochanter (13 RCTs); intertrochanter and ward triangle (WT; 10 RCTs each); total spine (TS; 4 RCTs); whole femurs (WF; 3 RCTs); and arms, femoral trochanter, legs, and pelvis (2 RCTs each). Single RCTs assessed BMD at additional forearm sites (distal radius and ulna 1/10, forearm 33% radius, radius 1/3 of styloid process, total radius, ultradistal radius, whole forearms).

Bone turnover markers were reported as follows: OC (23 RCTs), DPD (18 RCTs), 25(OH)D (14 RCTs), CTx (16 RCTs), NTx (12 RCTs), BAP (19 RCTs), PTH (12 RCTs), PINP (10 RCTs), IGF-1 (8 RCTs), and pyridinoline (6 RCTs).

### 3.3. Network Meta-Analysis of the Impact of Interventions on Bone Health

As shown in Table 1, the included RCTs (1995–2022) involved 6284 healthy women. The mean age of the participants was 54.2 years, with a range from 19.6 to 74.2 years. The average BMI was 25.1 kg/m^2^, ranging from 20.4 to 32.2 kg/m^2^. The duration of the interventions averaged 11.3 months.

#### 3.3.1. Food-Class Level: Effects of Dairy and Soybean Products on Bone Health Outcomes

**Figure 2:**
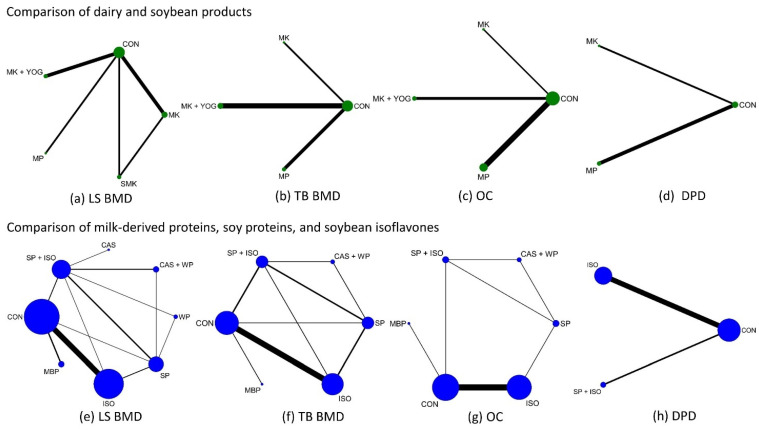
Network plots for lumbar spine (LS) BMD, total body (TB) BMD, osteocalcin (OC), and deoxypyridinoline (DPD). Each node symbolizes an intervention, and each connecting line represents a direct comparison between two interventions. The node size and the line thickness reflect the number of participants evaluating each intervention and comparison, respectively. CON: control (placebo/no treatment); SP: soy protein; ISO: isoflavone; SP + ISO: soy protein + isoflavone; MBP: milk basic protein; CAS: casein; WP: whey protein; CAS + WP: casein + whey protein; MK: milk; MK + YOG: milk + yogurt; MP: milk powder; SMK: soymilk.

**Figure 3:**
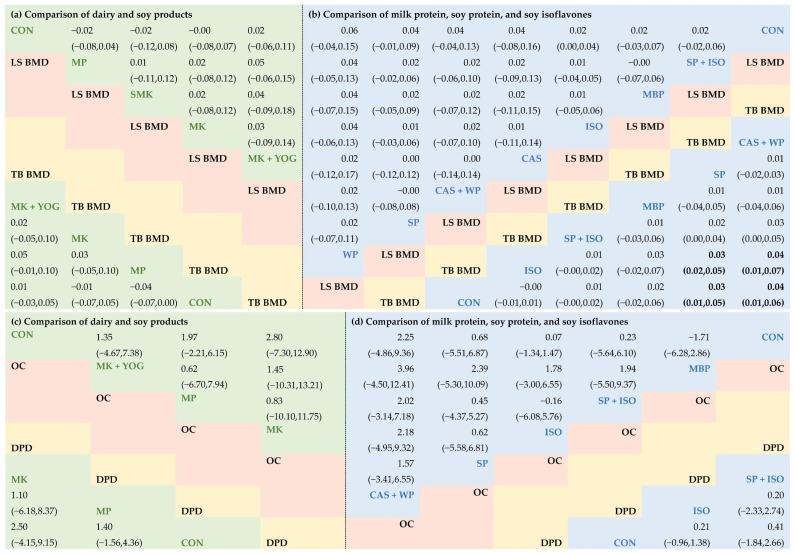
Comparative results of the network meta-analysis of the included studies with regard to LS BMD, TB BMD, and the bone turnover markers OC and DPD. The green area on the left represents the comparison outcomes of the food-class level (**a**,**b**), while the blue area on the right illustrates the comparison results of the bioactive-component level (**c**,**d**). CON: control (placebo/no treatment); SP: soy protein; ISO: isoflavone; SP + ISO: soy protein + isoflavone; MBP: milk basic protein; CAS: casein; WP: whey protein; CAS + WP: casein + whey protein; MK: milk; MK + YOG: milk + yogurt; MP: milk powder; SMK: soymilk.

The NMA on LS BMD included 5 RCTs (*n* = 458) evaluating five intervention pairs, forming one closed loop (Figure 2a and Table S6). The outcomes of both direct and indirect assessments within the NMA are presented in Figure 3a and Figure S15. No dairy interventions significantly differed from control, though milk + yogurt ranked numerically highest (SUCRA: 73.1%) (Table 2). The LS BMD analysis showed good global consistency (*p* = 0.325), supporting the use of the consistency model (Figure S4). Closed-loop analysis revealed no local inconsistencies (all *p* > 0.05; IF = 0.078; Table S12). Table S6 presents the local inconsistencies from “direct versus indirect” comparisons in five intervention pairs, using the side-splitting model.

For TB BMD, the NMA of six RCTs (*n* = 834) assessed three intervention pairs without closed loops (Figure 2b and Table S7). No dairy interventions significantly differed from control, though milk + yogurt ranked numerically highest (SUCRA: 81.9%) (Table 2). No closed loops were detected.

Analyses of bone turnover markers revealed no significant effects for dairy interventions on OC (7 RCTs, *n* = 944) or DPD (3 RCTs, *n* = 295), yet milk consistently exhibited the highest SUCRA values (OC: 62.3%; DPD: 69.1%) (Figure 2c,d and Figure 3a,b, Table 2).

#### 3.3.2. Bioactive-Component Level: Effects of Milk-Derived Proteins, Soybean Proteins, and Soybean Isoflavones on Bone Health Outcomes

At the bioactive-component level, LS BMD analysis (22 RCTs, *n* = 2159) evaluated twelve intervention pairs with six closed loops (Figure 2e and Table S6). No interventions significantly outperformed control, but whey protein (SUCRA: 74.4%), soybean protein (SUCRA: 43.9%), and casein + whey protein (SUCRA: 60.8%) ranked the highest (Figure 3c, Table 2). LS BMD exhibited good global consistency (*p* = 0.255), supporting the consistency model (Figure S5). Loop inconsistency was detected in three loops (*p* < 0.05; Table S12). Table S6 presents the local inconsistency from “direct versus indirect” comparisons in twelve intervention pairs using the side-splitting model.

For TB BMD (12 RCTs, *n* = 1220), the NMA evaluated nine intervention pairs with five closed loops (Figure 2f and Table S7). Casein + whey protein significantly increased BMD versus control (MD: 0.04 g/cm^2^, 95% CI: 0.01–0.06) and soy isoflavones (MD: 0.04 g/cm^2^, 95% CI: 0.01–0.07). Soybean protein also exceeded control (MD: 0.03 g/cm^2^, 95% CI: 0.01–0.05) and isoflavones alone (MD: 0.03 g/cm^2^, 95% CI: 0.02–0.05). Casein + whey protein ranked highest (SUCRA: 86.3%), followed by soybean protein (SUCRA: 79.7%) and MBP (SUCRA: 63.2%). (Figure 3c, Table 2). TB BMD showed good global consistency (*p* = 0.169), supporting the consistency model (Figure S7). No loop inconsistency was found (all *p* > 0.05; IF range: 0.005–0.053; Table S13). Table S7 presents the local inconsistencies from the “direct versus indirect” comparisons in nine intervention pairs using the side-splitting model.

Among bone turnover markers, casein + whey protein showed the highest efficacy for OC (SUCRA: 75.9%; 15 RCTs, *n* = 918), while soybean protein + isoflavone ranked highest for DPD (SUCRA: 61.0%; 13 RCTs, *n* = 1168), though no interventions significantly differed from control (Figure 2g,h and Figure 3d; Table 2, Tables S8 and S9). OC analysis indicated good global consistency (*p* = 0.322), supporting the consistency model (Figure S9). Loop inconsistency was detected in one loop (*p* < 0.05; Table S14). Table S8 presents the local inconsistency from “direct versus indirect” comparisons in seven intervention pairs using the side-splitting model. No closed loops were detected in the DPD analysis.

### 3.4. Subgroup Analysis and Sensitivity Analysis

Subgroup analyses were conducted based on baseline BMI (<24 vs. ≥24 kg/m^2^) to explore its role as a potential effect modifier, as suggested in the Methods. The results are detailed in Tables S16–S18. Notably, in participants with a BMI ≥ 24 kg/m^2^, the ranking of bioactive components was highly consistent with the primary findings from the overall analysis. The combination of casein + whey protein (SUCRA: 90.3%) and soybean protein (SUCRA: 80.4%) remained the most effective interventions for improving TB BMD, and whey protein (SUCRA: 72.5%) remained the most effective for improving LS BMD, underscoring the robustness of these findings in women with higher BMI. In contrast, a different pattern emerged in the lower BMI subgroup (<24 kg/m^2^), where MBP demonstrated the highest efficacy for both LS and TB BMD (SUCRA: 90.3% and 85.3%, respectively). For bone turnover markers, the rankings also varied between subgroups. Isoflavone ranked highest for OC in the lower BMI group, while casein + whey protein and isoflavone were top-ranked for OC and DPD, respectively, in the higher BMI group.

To assess the robustness of the primary network against local inconsistencies, sensitivity analyses were performed by excluding studies contributing to inconsistent loops. As shown in Tables S19–S21, the exclusion of these data did not materially alter the effect estimates or the relative SUCRA rankings, confirming the stability and reliability of our main conclusions.

### 3.5. Risk-of-Bias Assessment

The results of the RCT risk-of-bias evaluation are shown in Figures S1 and S2. Of the sixty RCTs, the majority of studies were classified as “low risk” in the categories “Missing outcome data” (49 studies, 81.7%), “Selection of the reported result” (48 studies, 80%), “Measurement of the outcome” (47 studies, 78.3%), and “Deviations from intended interventions” (42 studies, 70.0%). Conversely, a significant number of studies raised “some concerns” in the “Randomization process” category (33 studies, 55%).

### 3.6. Publication Bias

Publication bias was assessed for outcomes with sufficient data (≥10 studies) using funnel plots and formal statistical testing via Egger’s test. As shown in Figure S3, the funnel plots for LS, TB, OC, and DPD all demonstrated approximate symmetry. This visual interpretation was corroborated by Egger’s test, which yielded non-significant *p*-values (all *p* > 0.05), indicating no substantial evidence of publication bias.

## 4. Discussion

This NMA study represents the first comprehensive evaluation of the effects of both food-level (dairy vs. soy products) and component-level (specific proteins and isoflavones) interventions on bone health in healthy women. By synthesizing direct and indirect evidence from 60 RCTs involving 6284 participants, we established two principal findings. First, at the food level, no dairy or soy product was found to have a significant effect in improving BMD or bone turnover markers compared to control, although milk + yogurt or milk ranked numerically highest. Second, at the bioactive-component level, whey protein—either alone or combined with casein—and soy protein emerged as the highest-ranked interventions. Furthermore, subgroup analysis suggests that BMI may influence the effect of certain components on bone health, with MBP demonstrating more pronounced benefits in individuals with a lower BMI. These findings provide nuanced insights for developing targeted dietary strategies to maintain bone health.

The lack of statistically significant differences at the food level, contrary to some previous hypotheses, may be attributed to several factors. The inherent heterogeneity in the forms of dairy and soy products (e.g., liquid milk vs. powder, fortified vs. non-fortified, varying fat content) likely diluted the specific effects of any single food category. Furthermore, the control groups in many studies may have had relatively adequate baseline nutrient intakes, diminishing the observable benefit of additional dietary interventions. Despite the absence of statistical significance, the consistently highest ranking of the milk + yogurt combination is noteworthy. This may be due to a synergistic effect: the components in milk (calcium, protein, vitamin D) are complemented by the probiotics and fermentation products in yogurt, which may enhance calcium bioavailability, reduce systemic inflammation, and positively modulate the gut–bone axis, potentially leading to more favorable bone remodeling outcomes [12,15,105]

As essential bioactive ingredients that benefit bone health, calcium and vitamin D have long been regarded as crucial [15]. However, recent studies have indicated that these two nutrients may not confer the anticipated substantial benefits [24,25,26]. Conversely, the role of high-quality protein in maintaining bone health has increasingly garnered attention [27,28,29]. Previous systematic reviews have generally compared animal and plant proteins without differentiating between specific protein types [31]. In our component-level NMA, whey protein appeared to be central to dairy’s osteoprotective effects. Its highest ranking for LS BMD and superior performance for TB BMD when combined with casein underscore its biological potency. Mechanistically, whey’s rapid digestibility and rich essential amino acid composition enhance anabolic signaling pathways, while its bioactive peptides may stimulate osteoblast activity through insulin-like growth factor-1 upregulation and activation of the Runx2/GSK-3β/Nrf2 pathway [106,107,108]. Interestingly, soy protein ranked second in efficacy for multiple bone health outcomes, outperforming isolated casein or milk basic protein. Animal models corroborate this hierarchy, showing soy-fed subjects exhibit better bone metrics than casein-fed counterparts, potentially due to isoflavone-enhanced osteogenic activity [109]. However, our analysis revealed that isoflavones alone showed minimal efficacy, suggesting that their action requires the synergistic presence of soy protein rather than functioning as isolated agents. Furthermore, the biological effects of isoflavones are known to vary significantly due to metabolic differences, particularly between individuals who can produce equol and those who cannot [110]. This variability in bioavailability likely contributes to the lack of observed significant effect of isoflavones in our overall analysis. Baseline dietary calcium/protein intake may also influence the intervention outcomes. Previous studies have indicated that baseline levels of dietary calcium and protein can modulate each other’s beneficial effects on bone health. Specifically, in postmenopausal women, the dietary calcium-to-protein ratio has potential to be a critical factor in bone health interventions [111,112]. Additionally, there are notable regional disparities worldwide in the intake of key nutrients, including calcium [113]. However, as none of the original studies included in this network meta-analysis reported data on equol producer status, and comprehensive data on nutritional intake were unavailable, we were unable to conduct further subgroup analyses to evaluate these hypotheses, restricting the generalizability of our conclusions.

An intriguing observation pertains to the discordance between BMD outcomes and bone turnover markers. While multiple interventions significantly improved BMD, their effects on OC and DPD were inconsistent. This discrepancy may reflect several factors. Studies measuring turnover markers typically employed shorter intervention periods (often <6 months) compared to BMD trials, potentially capturing only acute-phase remodeling rather than structural adaptation. Additionally, biochemical markers exhibit greater analytical variability and diurnal fluctuations than dual-energy X-ray absorptiometry measurements. Furthermore, effective interventions may normalize elevated bone resorption without suppressing formation markers, maintaining metabolic equilibrium while progressively improving bone density.

Our subgroup analysis based on BMI revealed important differences in the efficacy of bioactive components. In women with a lower BMI (<24 kg/m^2^), MBP demonstrated the highest efficacy for improving BMD. This suggests that leaner individuals, who may have a more efficient anabolic response and lower inflammatory burden, might benefit more from specific bioactive peptides like those found in MBP. Conversely, in women with higher BMI (≥24 kg/m^2^), the whey protein and the combination of whey protein and casein were most effective for LS and TB BMD. This could be attributed to whey protein’s well-documented anti-inflammatory and antioxidant properties, which may help mitigate the chronic low-grade inflammation associated with obesity—a known contributor to bone resorption and impaired bone remodeling [7,108]. These findings suggest that body composition may be an important effect modifier when recommending protein supplements for bone health.

The methodological approach of this study presents notable advantages compared to previous systematic reviews. By employing a dual-level NMA framework, we simultaneously addressed food-level and component-level questions. The SUCRA values offer clinically meaningful insights into the relative efficacy of various interventions. At the practical level, findings at the food level can inform dietary recommendations (e.g., recommending powdered dairy products for lactose-tolerant women), whereas component-level results contribute valuable guidance for the development of functional foods (e.g., whey-fortified products tailored for bone health).

This study also has important limitations warranting consideration when interpreting these findings. First, many intervention comparisons relied solely on indirect evidence due to the scarcity of head-to-head trials. This, unfortunately, led to several local inconsistencies in certain intervention pairs, potentially undermining confidence in those specific estimates. To address this, we conducted sensitivity analyses by excluding the inconsistent loops, which confirmed that the overall SUCRA rankings and effect estimates remained stable. Furthermore, no global inconsistency was detected across the networks, which together support the robustness of our primary conclusions. Second, more than half (55%) of the trials raised some concerns regarding the randomization process according to the ROB2 assessment, potentially compromising the robustness of the evidence. Third, considerable variability was observed in intervention characteristics—including differences in dosage, formulation matrices, and baseline nutrient status—which may contribute to clinical heterogeneity. Fourth, our analysis was limited to surrogate markers of bone health, specifically BMD and biochemical turnover markers (e.g., OC, DPD), as data on microarchitectural properties and fracture outcomes were unavailable in the included trials. Although improvements in these markers are biologically plausible and widely used, their ability to predict fracture risk is limited and non-linear. For example, BMD changes only partially explain anti-fracture effects in interventions. Thus, while certain interventions improved bone density and metabolism, their actual clinical benefit for fracture prevention remains uncertain. Fifth, the analysis could not stratify effects based on key physiological variables such as menopausal status or genetic polymorphisms influencing nutrient metabolism, limiting its applicability for personalized interventions. Finally, generalizability to populations with pre-existing bone pathologies remains uncertain, as the analysis included only healthy women.

Future research should address these gaps using several approaches. Several intervention pairs that currently have limited evidence for direct comparisons are worth further investigation. Clinical trials should prioritize long-term intervention periods with fracture outcomes or incorporate three-dimensional bone imaging endpoints to more directly assess bone quality and strength. Direct comparator trials evaluating whey protein against soy protein, or milk powder versus fortified soymilk, would strengthen the evidence base. Mechanistic human studies examining whey’s effects on bone-related signaling pathways would further elucidate its osteoprotective actions.

## 5. Conclusions

In conclusion, our findings suggest that at the food level, while no specific dairy or soy product significantly outperformed control, the combination of milk and yogurt showed the highest ranking potential for benefiting bone health. At the bioactive-component level, whey protein (alone or combined with casein) and soybean protein are the most effective interventions for enhancing BMD outcomes. The efficacy of these components may be influenced by BMI, highlighting the potential for personalized nutrition strategies. These results offer evidence-based guidance for dietary choices supporting women’s bone health, suggesting the possible benefits of milk + yogurt consumption and identifying whey protein as a key functional component. Soybean protein presents a viable alternative for non-dairy consumers. Future research should focus on direct comparisons of these high-ranking interventions and investigate their effects on bone microarchitecture and fracture risk.

## Figures and Tables

**Table 1:** The characteristics of the included RCTs.

Study (Year)	Country	Intervention Period	*n*	Age (Years)	BMI (kg/m^2^)	Comparison	Outcomes
				(M ± SD)			
1 Albertazzi (2005) [46]	UK	6 weeks	100	53.5 ± 3.0	27.0 ± 6.1	Isoflavone (genistein, 90 mg) vs. placebo	OC, CTx
2 Alekel (2000) [47]	USA	24 weeks	69	50.2 ± 3.9	24.0 ± 3.2	Soy protein (40 g/d) vs. whey protein (40 g/d) vs. soy protein (40 g/d) with isoflavone (80.4 mg/d)	BMD (LS)
3 Anderson (2002) [48]	USA	12 months	28	23.9 ± 1.0	21.4 ± 3.8	Isoflavone (90 mg/d) vs. soy protein	BMD (TB, FN, LS, trochanter, intertrochanter, WT)
4 Aoe (2005) [49]	Japan	6 months	27	50.5 ± 3.0	21.6 ± 2.9	MBP (40 mg/d) vs. placebo	OC, NTx
5 Arjmandi (2003) [50]	USA	3 months	42	62.1 ± 4.9	32.2 ± 7.9	Soy protein (40 g/d) vs. milk protein (casein-whey protein, 40 g/d)	BAP, DPD, IGF-1
6 Arjmandi (2005) [51]	USA	12 months	62	54.3 ± 5.7	28.0 ± 5.3	Soy protein (25 g/d) with isoflavone (60 mg/d) vs. placebo	BMD (TB, LS, TH), OC, BAP, DPD, IGF-1
7 Atteritano (2009) [52]	Italy	2 years	138	52.5 ± 2.1	24.5 ± 3.5	Isoflavone (genistein, 54 mg/d) vs. placebo	BMD (FN, LS)
8 Bonjour (2008) [53]	France	6 weeks	60	59.5 ± 3.3	23.7 ± 3.4	Semi-skimmed milk (500 mL/d) vs. no treatment	OC, BAP, PINP, CTx, PTH, IGF-1, 25(OH)D
9 Bonjour (2018) [54]	France	16 weeks	133	61.5 ± 5.0	24.6 ± 2.9	Yogurts (125 g/d or 250 g/d) vs. no treatment	25(OH)D
10 Brink (2008) [55]	Netherlands, Italy, France	53 weeks	237	53.0 ± 3.0	24.5 ± 2.1	Isoflavone vs. placebo	BMD (TB, LS), BAP, PINP, DPD, Pyr, PTH, 25(OH)D
11 Brooks (2004) [56]	Canada	16 weeks	28	53.4 ± 3.2	27.4 ± 5.3	Soy (25 g/d) with isoflavones (41.9 mg/d) vs. placebo	BAP, DPD
12 Chee (2003) [57]	China	24 months	173	58.8 ± 3.5	23.8 ± 3.5	High-calcium skimmed milk powder (50 g/d) vs. no treatment	BMD (TB, FN, LS, TH), OC, DPD, PTH, 25(OH)D
13 Chen (2003) [58]	China	1 year	175	54.2 ± 3.1	24.0 ± 3.5	Isoflavones (40 mg/d or 80 mg/d) vs. placebo	BMD (TB, FN, LS, TH, trochanter, intertrochanter)
14 Chilibeck (2013) [59]	Canada	2 years	149	55.7 ± 6.9	27.1 ± 3.9	Isoflavone (165 mg/d) vs. placebo	BMD (TB, FN, LS, TH, trochanter, WT)
15 Cleghorn (2001) [60]	Australia	1 year	72	52.0 ± 3.0	26.1 ± 5.4	Calcium-fortified milk (3 L/wk) vs. no treatment	DPD
16 Choquette (2011) [61]	Canada	6 months	45	59.0 ± 5.0	30.1 ± 2.8	Isoflavones (70 mg/d) vs. placebo	BMD (TB, FN, LS, TH, trochanter, WT)
17 Dalais (2003) [62]	Australia	3 months	78	60.0 ± 6.2	25.4 ± 4.7	Soy protein (40 g/d) with isoflavones (118 mg/d) vs. casein (40 g/d)	DPD, Pyr
18 Gallagher (2004) [63]	USA	15 months	50	55.4 ± 4.6	26.4 ± 5.3	soy protein (40 g/d) vs. soy protein (40 g/d) with isoflavones (52 mg/d or 96 mg/d)	BMD (FN, LS, trochanter), OC, NTx
19 Green (2002) [64]	New Zealand	4 weeks	50	67.6 ± 6.7	27.5 ± 4.5	High-calcium skim milk powder (50 g/d) vs. placebo	PINP, DPD, NTx, CTx, PTH
20 Gui (2012) [65]	China	18 months	98	56.3 ± 4.2	24.5 ± 3.0	Calcium-fortified milk (250 mL/d) vs. soy milk (250 mL/d) vs. no treatment	BMD (FN, LS, TH)
21 Harkness (2004) [66]	USA	6 months	38	70.6 ± 6.3	25.9 ± 3.5	Soy isoflavone (110 mg/d) vs. placebo	BMD (TS, trochanter, intertrochanter), OC, BAP
22 Huang (2006) [67]	China	12 months	42	52.4 ± 2.8	23.5 ± 2.6	Soy isoflavone (100 mg/d or 200 mg/d) vs. no treatment	BMD (FN, femoral trochanter, LS, WT), BAP, DPD, NTx
23 Ilich (2019) [68]	USA	6 months	60	55.8 ± 4.3	31.5 ± 5.1	Low-fat dairy foods (cheeses, milk, yogurt, pudding, low-fat ice cream, 4–5 servings/d) vs. placebo	BMD (TB, FN, LS, WF, radius 1/3 of styloid process, whole forearms), OC, NTx, CTx, PTH, 25(OH)D
24 Kenny (2009) [69]	USA	1 year	97	73.1 ± 5.9	28.3 ± 5.4	Soy protein (20 g/d) vs. soy isoflavones (105 mg/d) vs. placebo	BMD (TB, FN, LS, WF, trochanter, WT, forearm 33% radius, ultradistal radius, total radius), BAP, NTx
25 Kreijkamp-Kaspers (2004) [70]	Netherlands	12 months	175	66.6 ± 4.7	26.2 ± 3.8	Soy protein (25.6 g/d) with isoflavones (99 mg/d) vs. 25.6 g of total milk protein (casein-whey protein)	BMD (LS, TH, trochanter, intertrochanter, WT), BAP
26 Kruger (2006) [71]	New Zealand	16 weeks	55	27.1 ± 4.8	24.1 ± 2.9	High calcium skim milk powder (50 g/d) vs. no treatment	OC, PINP, CTx, PTH, IGF-1, 25(OH)D
27 Kruger (2010) [72]	South East Asia	16 weeks	113	57.5 ± 4.4	24.8 ± 3.8	High-calcium vitamin D fortified milk powder (60 g/d) vs. placebo	OC, PINP, CTx, PTH
28 Kruger (2012) [73]	China	12 weeks	58	62.1 ± 4.2	25.7 ± 2.3	High-calcium vitamin D fortified milk (two servings/d) vs. placebo	PINP, CTx, PTH, 25(OH)D
29 Lau (2001) [74]	China	24 months	185	56.7 ± 1.7	26.2 ± 3.8	High calcium, low-fat, low-lactose milk powder (50 g/d) vs. no treatment	BMD (TB, FN, LS, TH, intertrochanter)
30 Lau (2002) [75]	China	3 years	197	56.9 ± 1.7	24.1 ± 4.1	High calcium, low-fat, low-lactose milk powder (50 g/d) vs. no treatment	BMD (TB, FN, LS, TH, intertrochanter)
31 Lee (2017) [76]	Korea	12 weeks	84	53.6 ± 3.4	——	Isoflavones (70 mg/d) vs. placebo	OC, BAP, DPD, NTx, CTx
32 Levis (2011) [77]	USA	24 months	177	52.5 ± 3.3	26.3 ± 3.3	Isoflavones (200 mg/d) vs. placebo	BMD (FN, LS, TH), NTx, 25(OH)D
33 Liu (2020) [78]	China	6 months	270	57.9 ± 5.0	——	Soy flour (40 g/d) vs. low-fat milk powder (40 g/d)	OC, BAP, PINP, CTx, 25(OH)D
34 Lydeking-Olsen (2004) [14]	Denmark	2 years	45	57.1 ± 7.6	23.9 ± 3.8	Soy milk (500 mL/d) with isoflavone (76.0 mg/d) vs. soy milk (500 mL/d)	BMD (LS)
35 Manios (2007) [79]	Greece	12 months	55	60.9 ± 4.8	29.4 ± 4.8	Dairy products fortified with calcium and vitamin D3 (3 portions/d, one portion equals 250 mL milk and 200 g yogurt) vs. no treatment	BMD (TB, LS, TS, pelvis, arms, legs), OC, CTx, PTH, IGF-1, 25(OH)D
36 Marini (2007) [80]	Italy	2 years	304	54.5 ± 3.1	25.0 ± 3.8	Isoflavone (genistein, 54 mg/d) vs. placebo	BMD (FN, LS), BAP, DPD, Pyr, IGF-1, 25(OH)D
37 Morabito (2002) [81]	Italy	12 months	60	51.5 ± 3.5	24.0 ± 2.5	Isoflavone (genistein, 54 mg/day) vs. placebo	BMD (FN, LS, WT), OC, BAP, DPD, PTH, 25(OH)D
38 Mori (2004) [82]	Japan	4 weeks	43	40–63	22.3 ± 2.0	Isoflavones (40 mg/d) vs. placebo	OC, DPD
39 Mori (2004) [83]	Japan	24 weeks	70	49.7 ± 4.8	22.0 ± 2.7	Isoflavones (100 mg/d) vs. placebo	BMD (TB)
40 Moschonis (2010) [84]	Greece	30 months	66	59.8 ± 4.7	28.9 ± 5.1	Low-fat dairy products fortified with Ca and vitamin D3 (3 portions/d, milk and yogurt) vs. no treatment	BMD (TB, TS, pelvis, arms, legs)
41 Norton (2022) [85]	Ireland	24 weeks	67	62.8 ± 6.0	26.2 ± 4.3	Milk protein (casein-whey protein) vs. placebo	PINP, CTx
42 Prince (1995) [86]	Australia	2 years	84	63.0 ± 4.0	——	Milk powder (208 mL/d) vs. placebo	BMD (FN, trochanter, intertrochanter)
43 Radhakrishnan (2009) [87]	India	6 months	85	48.9 ± 6.4	25.5 ± 4.4	Soy protein (25 g/d) with isoflavone (75 mg/d) vs. casein protein (25 g/d)	BMD (FN, LS)
44 Sathyapalan (2017) [88]	UK	6 months	200	52.0 ± 4.5	25.5 ± 4.5	Soy protein (15 g/d) with isoflavones (66 mg/d) vs. soy protein (15 g/d)	PINP, CTx
45 Spence (2005) [89]	New Zealand	12 weeks	45	57.0 ± 6.0	29.0 ± 7.0	Soy protein (40 g/d) with isoflavones vs. soy protein (40 g/d) vs. milk protein (casein-whey protein, 40 g/d)	OC, BAP, NTx, PTH, 25(OH)D
46 Tai (2012) [90]	China	96 weeks	396	55.8 ± 3.8	22.9 ± 2.6	Isoflavone (300 mg/d) vs. placebo	BMD (LS), BAP, NTx
47 Tousen (2011) [91]	Japan	12 months	93	53.8 ± 3.7	22.0 ± 3.0	Isoflavone (S-equol, 2 mg/d or 6 mg/d or 10 mg/d) vs. placebo	BMD (TB, FN, LS, TH, trochanter, intertrochanter, WT), OC, BAP, DPD, NTx
48 Turhan (2008) [92]	Turkey	6 months	90	53.9 ± 7.1	27.0 ± 3.1	Isoflavone (genistein, 60 mg/d) vs. placebo	OC, CTx
49 Uenishi (2007) [93]	Japan	6 months	35	21.0 ± 1.0	20.8 ± 2.3	MBP (40 mg/d) vs. placebo	BMD (LS), OC, NTx, CTx
50 Uesugi (2002) [94]	Japan	4 weeks	23	51.4 ± 5.9	22.6 ± 2.8	Isoflavones (61.8 mg/d) vs. placebo	OC, DPD, Pyr
51 Uesugi (2003) [95]	Japan	3 months	21	51.5 ± 5.3	22.5 ± 2.7	Isoflavones (61.8 mg/d) vs. placebo	BMD (LS), Pyr
52 Vupadhyayula (2009) [96]	USA	24 months	157	63.6 ± 4.5	26.2 ± 4.0	Soy protein (25 g/d) vs. casein and whey protein (25 g/d) vs. soy protein (25 g/d) with isoflavones (90 mg/d)	BMD (TB, FN, femoral trochanter, LS, WF)
53 Wangen (2000) [97]	USA	12 weeks	51	57.1 ± 5.9	25.2 ± 3.7	Isoflavones (65 mg/d or 130 mg/d) vs. placebo	OC, BAP, DPD, CTx, IGF-1
54 Woo (2007) [98]	China	24 months	408	28.0 ± 8.0	20.4 ± 3.6	Milk powder (two sachets/d) vs. no treatment	BMD (TB, TH, TS), OC, BAP, PINP, NTx, CTx, PTH, 25(OH)D
55 Wu (2006) [99]	Japan	12 months	66	54.4 ± 2.9	21.1 ± 2.3	Isoflavone (75 mg/d) vs. placebo	BMD (TB, FN, LS, TH, trochanter, WT), OC, BAP, DPD
56 Wu (2007) [100]	Japan	12 months	54	54.4 ± 2.9	21.1 ± 2.5	Isoflavone (75 mg/d) vs. placebo	BMD (TB, FN, LS, TH, trochanter, intertrochanter, WT)
57 Yamori (2002) [101]	Japan	10 weeks	40	53.2 ± 3.5	25.8 ± 3.7	Isoflavones (37.3 mg/d) vs. placebo	DPD, Pyr
58 Ye (2006) [102]	China	6 months	84	52.3 ± 3.3	22.7 ± 2.4	Soy isoflavones (84 mg/d or 126 mg/d) vs. placebo	BMD (FN, LS, TH, trochanter, intertrochanter), OC, BAP, DPD
59 Zhu (2011) [103]	Australia	2 years	196	74.2 ± 2.7	26.7 ± 3.9	Whey protein (30 g/d) vs. placebo	BMD (FN, TH), IGF-1
60 Zou (2009) [104]	China	8 months	81	19.6 ± 0.6	20.4 ± 1.8	MBP (40 mg/d) vs. whole milk (250 mL/d) vs. no treatment	BMD (TB, LS, Dist R + U 1/10)

BAP: bone-specific alkaline phosphatase; BMD: bone mineral density; BMI: body mass index; CTx: *C*-terminal telopeptide of type I collagen; DPD: deoxypyridinoline; FN: femoral neck; IGF-1: Insulin-like growth factor 1; LS: lumbar spine; MBP: milk basic protein; NTx: *N*-terminal telopeptide of type I collagen; OC: osteocalcin; PINP: procollagen type I *N*-terminal propeptide; PTH: parathyroid hormone; Pyr: pyridinoline; SD: standard deviation; TB: total body; TH: total hip; TS: total spine; WF: whole femurs; WT: ward triangle; [25(OH)D]: 25-hydroxyvitamin D.

**Table 2:** Intervention rankings using surface under the cumulative ranking (SUCRA) values.

Rank	LS BMD		TB BMD		OC		DPD	
	Intervention	SUCRA (%)	Intervention	SUCRA (%)	Intervention	SUCRA (%)	Intervention	SUCRA (%)
Comparison of dairy and soy products								
1	MK + YOG	73.1	MK + YOG	81.9	MK	62.3	MK	69.1
2	CON	55.8	CON	60.7	MP	61.2	MP	60.2
3	MK	53.0	MK	46.5	MK + YOG	49.5	CON	20.7
4	SMK	38.7	MP	1.1	CON	27.0		
5	MP	29.4						
Comparison of milk protein, soy protein, and soy isoflavones								
1	WP	74.4	CAS + WP	86.3	CAS + WP	75.9	SP + ISO	61.0
2	SP	63.9	SP	79.7	SP	54.9	ISO	53.4
3	CAS + WP	60.8	MBP	63.2	ISO	49.2	CON	35.6
4	CAS	56.4	SP + ISO	43.4	SP + ISO	48.2		
5	ISO	48.7	CON	18.1	CON	47.6		
6	MBP	40.5	ISO	9.1	MBP	24.3		
7	SP + ISO	39.4						
8	CON	15.9						

DPD: deoxypyridinoline; LS: lumbar spine; OC: osteocalcin; SUCRA: surface under the cumulative ranking curve; TB: total body; CON: control (placebo/no treatment); SP: soy protein; ISO: isoflavone; SP + ISO: soy protein + isoflavone; MBP: milk basic protein; CAS: casein; WP: whey protein; CAS + WP: casein + whey protein; MK: milk; MK + YOG: milk + yogurt; MP: milk powder; SMK: soymilk.

## Data Availability

The data presented in the manuscript will be provided upon request by contacting the corresponding authors. The data are not publicly available due to ethical and participant privacy.

## Supplementary Materials

The following supporting information can be downloaded at: https://www.mdpi.com/article/10.3390/nu17172833/s1, Table S1: Search strategy in PubMed; Table S2: Search strategy in Cochrane Library; Table S3: Search strategy in Embase; Table S4: Search strategy in Web of Science; Table S5: Summary of included research outcomes; Table S6: Inconsistency test between direct and indirect treatment comparisons in mixed treatment comparison for LS BMD; Table S7: Inconsistency test between direct and indirect treatment comparisons in mixed treatment comparison for TB BMD; Table S8: Inconsistency test between direct and indirect treatment comparisons in mixed treatment comparison for OC; Table S9: Inconsistency test between direct and indirect treatment comparisons in mixed treatment comparison for DPD; Table S10: Inconsistency test between direct and indirect treatment comparisons in mixed treatment comparison for CTx; Table S11: Inconsistency test between direct and indirect treatment comparisons in mixed treatment comparison for P1NP; Table S12: Loop-specific heterogeneity of LS BMD; Table S13: Loop-specific heterogeneity of TB BMD; Table S14: Loop-specific heterogeneity of OC; Table S15: Comparative results of network meta-analysis of bone turnover markers CTx and P1NP in the included studies; Table S16: Comparative results of subgroup analysis in LS and TB BMD; Table S17: Comparative results of subgroup analysis in bone turnover markers OC and DPD; Table S18: Subgroup analysis of intervention rankings using surface under the cumulative ranking (SUCRA) values; Table S19: Comparative results of sensitivity analysis in LS and TB BMD (excluding MBP); Table S20: Comparative results of sensitivity analysis in bone turnover markers OC and DPD (excluding MBP); Table S21: Sensitivity analysis of intervention rankings using surface under the cumulative ranking (SUCRA) values (excluding MBP); Figure S1: Graph of Cochrane risk-of-bias assessment; Figure S2: Bias risk of the included studies; Figure S3: Publication bias assessed via funnel plots; Figure S4: Global inconsistency test for LS BMD of dairy products vs. soybean; Figure S5: Global inconsistency test for LS BMD of milk-derived protein vs. soy protein and isoflavone; Figure S6: Global inconsistency test for TB BMD of dairy products vs. soybean; Figure S7: Global inconsistency test for TB BMD of milk-derived protein vs. soy protein and isoflavone; Figure S8: Global inconsistency test for OC of dairy products vs. soybean; Figure S9: Global inconsistency test for OC of milk-derived protein vs. soy protein; Figure S10: Global inconsistency test for DPD of dairy products vs. soybean; Figure S11: Global inconsistency test for DPD of milk-derived protein vs. soy; Figure S12: Global inconsistency test for CTx of dairy products vs. soybean; Figure S13: Global inconsistency test for CTx of milk-derived protein vs. soy; Figure S14: Global inconsistency test for PINP of dairy products vs. soybean; Figure S15: Effect size for LS BMD of dairy vs. soybean using forest plots; Figure S16: Effect size for LS BMD of milk-derived protein vs. soy protein and isoflavone using forest plots; Figure S17: Effect size for TB BMD of dairy products using forest plots; Figure S18: Effect size for TB BMD of milk-derived protein vs. soy protein and isoflavone using forest plots; Figure S19: Effect size for OC of dairy products using forest plots; Figure S20: Effect size for OC of milk-derived protein vs. soy protein and isoflavone using forest plots; Figure S21: Effect size for DPD of dairy products using forest plots; Figure S22: Effect size for DPD of soy protein vs. isoflavone using forest plots; Figure S23: Effect size for CTx of dairy products using forest plots; Figure S24: Effect size for CTx of milk-derived protein vs. soy isoflavone using forest plots; Figure S25: Effect size for P1NP of dairy products using forest plots; Figure S26: Ranking diagram of each intervention for lumbar spine (LS) BMD and total body (TB) BMD; Figure S27: Ranking diagram of each intervention for osteocalcin (OC) and deoxypyridinoline and (DPD); Figure S28: Ranking diagram of each intervention for *C*-terminal telopeptide of type I collagen (CTx) and procollagen type I *N*-terminal propeptide (P1NP); Figure S29: Network plots for *C*-terminal telopeptide of type I collagen (CTx) and procollagen type I *N*-terminal propeptide (P1NP).

## Abbreviations

The following abbreviations are used in this manuscript:

BAP	bone-specific alkaline phosphatase
BMD	bone mineral density
CTx	*C*-terminal telopeptide of type I collagen
DPD	deoxypyridinoline
IGF-1	insulin-like growth factor 1
LS	lumbar spine
MBP	milk basic protein
MDs	mean differences
NMA	network meta-analysis
NTx	*N*-terminal telopeptide of type I collagen
OC	osteocalcin
PINP	procollagen type I *N*-terminal propeptide
PROSPERO	Prospective Register of Systematic Reviews
PTH	parathyroid hormone
Pyr	pyridinoline
RCTs	randomized controlled trials
SD	standard deviations
SE	standard errors
SUCRA	surface under the cumulative ranking curve
TB	total body
25(OH)D	25-hydroxyvitamin D
