# Supplementary material for: Comparing the Effects of Dairy and Soybean on Bone Health in Women: A Food- and Component-Level Network Meta-Analysis
Source: Nutrients. 2025 Aug 30;17(17):2833. doi: 10.3390/nu17172833 (PMC12430193; doi:10.3390/nu17172833)
Supplement: Supplementary file 1 [file nutrients-17-02833-s001.zip › nutrients-3821745-supplementary.pdf]

Supplementary materials

Table S1. Search strategy in PubMed.

Process	Search terms	Notes	No. of results
#1	"Dairy Products"[Mesh Terms] OR "Milk"[Mesh Terms] OR "Cultured Milk Products"[Mesh Terms] OR "Butter"[Mesh Terms] OR "Buttermilk"[Mesh Terms] OR "Cheese"[Mesh Terms] OR "Kefir"[Mesh Terms] OR "Koumiss"[Mesh Terms] OR "Yogurt"[Mesh Terms] OR "Milk Proteins"[Mesh Terms] OR "Whey Proteins"[Mesh Terms] OR "Caseins"[Mesh Terms] OR "Lactalbumin"[Mesh Terms] OR "Lactoglobulins"[Mesh Terms] OR "Lactoferrin"[Mesh Terms] OR "Osteopontin"[Mesh Terms] OR "Glycine max"[Mesh Terms] OR "Soybean Proteins"[Mesh Terms] OR "Isoflavones"[Mesh Terms] OR "Equol"[Mesh Terms]	Milk, dairy products, milk-derived proteins, soy protein, isoflavones<br><br>Subject term search	189,096
#2	"milk"[Title/Abstract] OR "dairy"[Title/Abstract] OR "yoghurt"[Title/Abstract] OR "Kefir"[Title/Abstract] OR "Koumiss"[Title/Abstract] OR "butter"[Title/Abstract] OR "cheese"[Title/Abstract] OR "whey"[Title/Abstract] OR "casein"[Title/Abstract] OR "lactalbumin"[Title/Abstract] OR "lactoglobulins"[Title/Abstract] OR "lactoferrin"[Title/Abstract] OR "osteopontin"[Title/Abstract] OR "glycomacropeptide"[Title/Abstract] OR "milk basic protein"[Title/Abstract] OR "soy"[Title/Abstract] OR "soybean"[Title/Abstract] OR "isoflavones"[Title/Abstract] OR "genistein"[Title/Abstract] OR "daidzein"[Title/Abstract] OR " equol"[Title/Abstract]	Milk, dairy products, milk-derived proteins, soy protein, isoflavones	329,125
#3	#1 OR #2		369,251
#4	"Bone and Bones"[MeSH Terms] OR "Bone Density"[MeSH Terms] OR "Fractures, Bone"[MeSH Terms] OR "Bone Remodeling"[MeSH Terms] OR "Osteogenesis"[MeSH Terms] OR "Bone Resorption"[MeSH Terms] OR "Osteoporosis, Postmenopausal"[MeSH Terms] OR "Osteoporosis"[MeSH Terms]	Bone<br>Bone Density<br>Fracture<br>Bone Remodeling<br>Bone Formation<br>Bone resorption<br>Osteoporosis	923,589

Process	Search terms	Notes	No. of results
#5	"bone"[Title/Abstract] OR "bone density"[Title/Abstract] OR "bone mineral density"[Title/Abstract] OR "BMD"[Title/Abstract] OR "bone mineral content"[Title/Abstract] OR "BMC"[Title/Abstract] OR "fracture"[Title/Abstract] OR "bone turnover"[Title/Abstract] OR "ossification"[Title/Abstract] OR "osteoporosis"[Title/Abstract] OR "osteopenia"[Title/Abstract] OR "demineralization"[Title/Abstract] OR "decalcification"[Title/Abstract]		1055,992
#6	#4 OR #5		1,545,247
#7	#3 AND #6		14,133
#8	("Randomized Controlled Trial"[Publication Type] OR "Controlled Clinical Trial"[Publication Type] OR "randomized"[Title/Abstract] OR "placebo"[Title/Abstract] OR "Clinical Trials as Topic"[MeSH Terms] OR "randomly"[Title/Abstract] OR "trial"[Title]) NOT ("Animals"[MeSH Terms] NOT "Humans"[MeSH Terms])		1,592,005
#9	#7 AND #8		1115

Table S2. Search strategy in Cochrane library.

Process	Search terms	Notes	No. of results
#1	MeSH descriptor: [Dairy Products] explode all trees OR MeSH descriptor: [Milk] explode all trees OR MeSH descriptor: [Cultured Milk Products] explode all trees OR MeSH descriptor: [Butter] explode all trees OR MeSH descriptor: [Buttermilk] explode all trees OR MeSH descriptor: [Cheese] explode all trees OR MeSH descriptor: [Kefir] explode all trees OR MeSH descriptor: [Koumiss] explode all trees OR MeSH descriptor: [Yogurt] explode all trees OR MeSH descriptor: [Milk Proteins] explode all trees OR MeSH descriptor: [Whey Proteins] explode all trees OR MeSH descriptor: [Caseins] explode all trees OR MeSH descriptor: [Lactalbumin] explode all trees OR MeSH descriptor: [Lactoglobulins] explode all trees OR MeSH descriptor: [Lactoferrin] explode all trees OR MeSH descriptor: [Osteopontin] explode all trees OR MeSH descriptor: [Glycine max] explode all trees OR MeSH descriptor: [Soybean Proteins] explode all trees OR MeSH descriptor: [Isoflavones] explode all trees OR MeSH descriptor: [Equol] explode all trees	Milk, dairy products, milk-derived proteins, soy protein, isoflavones<br><br>Subject term search	7503
#2	(milk):ti,ab,kw OR (dairy):ti,ab,kw OR (yoghurt):ti,ab,kw OR (Kefir):ti,ab,kw OR (Koumiss):ti,ab,kw OR (butter):ti,ab,kw OR (cheese):ti,ab,kw OR (whey):ti,ab,kw OR (casein):ti,ab,kw OR (lactalbumin):ti,ab,kw OR (lactoglobulins):ti,ab,kw OR (lactoferrin):ti,ab,kw OR (osteopontin):ti,ab,kw OR (glycomacropeptide):ti,ab,kw OR (milk basic protein):ti,ab,kw OR (soyb):ti,ab,kw OR (soybean):ti,ab,kw OR (isoflavones):ti,ab,kw OR (genistein):ti,ab,kw OR (daidzein):ti,ab,kw OR (equol):ti,ab,kw	Milk, dairy products, milk-derived proteins, soy protein, isoflavones	25370
#3	#1 OR #2		25603
#4	MeSH descriptor: [Bone and Bones] explode all trees OR MeSH descriptor: [Bone Density] explode all trees OR MeSH descriptor: [Fractures, Bone] explode all trees OR MeSH descriptor: [Bone Remodeling] explode all trees OR MeSH descriptor: [Osteogenesis] explode all trees OR MeSH descriptor: [Bone Resorption] explode all trees OR MeSH descriptor: [Osteoporosis, Postmenopausal] explode all trees OR MeSH descriptor: [Osteoporosis] explode all trees	Bone<br>Bone Density<br>Fracture<br>Bone Remodeling<br>Bone Formation<br>Bone resorption<br>Osteoporosis	33376
#5	(bone):ti,ab,kw OR (bone density):ti,ab,kw OR (bone mineral density):ti,ab,kw OR (BMD):ti,ab,kw OR (bone mineral content):ti,ab,kw OR (BMC):ti,ab,kw OR (fracture):ti,ab,kw OR (bone turnover):ti,ab,kw OR (ossification):ti,ab,kw OR (osteoporosis):ti,ab,kw OR (osteopenia):ti,ab,kw OR (demineralization):ti,ab,kw OR (decalcification):ti,ab,kw		87347
#6	#4 OR #5		97640
#7	#3 AND #6		1476

Table S3. Search strategy in Embase.

Process	Search terms	Notes	No. of results
#1	'dairy product'/exp OR 'milk'/exp OR 'fermented dairy product'/exp OR 'butter'/exp OR 'buttermilk'/exp OR 'cheese'/exp OR 'kefir'/exp OR 'koumiss'/exp OR 'yoghurt'/exp OR 'milk protein'/exp OR 'whey protein'/exp OR 'casein'/exp OR 'lactalbumin'/exp OR 'lactoglobulin'/exp OR 'lactoferrin'/exp OR 'osteopontin'/exp OR 'soybean'/exp OR 'soybean protein'/exp OR 'isoflavone'/exp OR 'equol'/exp	Milk, dairy products, milk-derived proteins, soy protein, isoflavones<br><br>Subject term search	238,069
#2	'milk':ab,ti OR 'dairy':ab,ti OR 'yoghurt':ab,ti OR 'kefir':ab,ti OR 'koumiss':ab,ti OR 'butter':ab,ti OR 'cheese':ab,ti OR 'whey':ab,ti OR 'casein':ab,ti OR 'lactalbumin':ab,ti OR 'lactoglobulin':ab,ti OR 'lactoferrin':ab,ti OR 'osteopontin':ab,ti OR 'glycomacropeptide':ab,ti OR 'milk basic protein':ab,ti OR 'soy':ab,ti OR 'soybean':ab,ti OR 'isoflavones':ab,ti OR 'genistein':ab,ti OR 'daidzein':ab,ti OR 'equol':ab,ti	Milk, dairy products, milk-derived proteins, soy protein, isoflavones	373,033
#3	#1 OR #2		425, 537
#4	'bone'/exp OR 'bone density'/exp OR 'fracture'/exp OR 'bone remodeling'/exp OR 'bone turnover'/exp OR 'osteolysis'/exp OR 'postmenopause osteoporosis'/exp OR 'osteoporosis'/exp	Bone<br>Bone Density<br>Fracture<br>Bone Remodeling<br>Osteolysis<br>Osteoporosis	1,450,121
#5	'bone':ab,ti OR 'bone density':ab,ti OR 'bone mineral density':ab,ti OR 'BMD':ab,ti OR 'bone mineral content':ab,ti OR 'BMC':ab,ti OR 'fracture':ab,ti OR 'bone turnover':ab,ti OR 'osteogenesis':ab,ti OR 'ossification':ab,ti OR 'osteoporosis':ab,ti OR 'osteopenia':ab,ti OR 'demineralization':ab,ti OR 'decalcification':ab,ti		1,398,101
#6	#4 OR #5		2,175,509
#7	#3 AND #6		21,200
#8	'randomized controlled trial'/exp OR 'controlled clinical trial'/exp OR 'clinical trials as topic'/exp		1,485,699
#9	'randomized':ab,ti OR 'placebo':ab,ti OR 'randomly':ab,ti OR 'trial':ti		1,821,068
#10	#8 OR #9		2,430,120
#11	'animal'/exp NOT 'human'/exp		6,174,157
#12	#10 NOT #11		2,230,338
#13	#7 AND #12		1511

Table S4. Search strategy in Web of science.

Process	Search terms	Notes	No. of results
#1	((((((((((((((((((TS=(dairy products)) OR TS=(milk)) OR TS=(cultured milk products)) OR TS=(butter)) OR TS=(buttermilk)) OR TS=(cheese)) OR TS=(Kefir)) OR TS=(Koumiss)) OR TS=(yogurt)) OR TS=(milk proteins)) OR TS=(whey proteins)) OR TS=(caseins)) OR TS=(lactalbumin)) OR TS=(lactoglobulins)) OR TS=(lactoferrin)) OR TS=(osteopontin)) OR TS=(soybeans)) OR TS=(soybean proteins)) OR TS=(isoflavones)) OR TS=(equol)	Milk, dairy products, milk-derived proteins, soy protein, isoflavones<br><br>Subject term search	555,872
#2	(((((((((((((((((((TS=(milk)) OR TS=(dairy)) OR TS=(yoghurt)) OR TS=(Kefir)) OR TS=(Koumiss)) OR TS=(butter)) OR TS=(cheese)) OR TS=(whey)) OR TS=(casein)) OR TS=(lactalbumin)) OR TS=(lactoglobulins)) OR TS=(lactoferrin)) OR TS=(osteopontin)) OR TS=(glycomacropeptide)) OR TS=(milk basic protein)) OR TS=soy) OR TS=(soybean)) OR TS=(isoflavones)) OR TS=(genistein)) OR TS=(daidzein)) OR TS=(equol)	Milk, dairy products, milk-derived proteins, soy protein, isoflavones	662,269
#3	#1 OR #2		666,221
#4	(((((((TS=(bone and bones)) OR TS=(bone density)) OR TS=(fractures, bone)) OR TS=(bone remodeling)) OR TS=(osteogenesis)) OR TS=(bone resorption)) OR TS=(osteoporosis, postmenopausal)) OR TS=(osteoporosis)	Bone<br>Bone Density<br>Fracture<br>Bone Formation<br>Bone Resorption<br>Bone Remodeling<br>Osteoporosis	1,126,307
#5	((((((((((((TS=(bone)) OR TS=(bone density)) OR TS=(bone mineral density)) OR TS=(BMD)) OR TS=(bone mineral content)) OR TS=(BMC)) OR TS=(fracture)) OR TS=(bone turnover)) OR TS=(ossification)) OR TS=(osteoporosis)) OR TS=(osteopenia)) OR TS=(demineralization)) OR TS=(decalcification)		1,691,067
#6	#4 OR #5		1,699,694
#7	#3 AND #6		20,422
#8	((((((TS=(randomized controlled trial)) OR TS=(controlled clinical trial)) OR TS=(randomized)) OR TS=(placebo)) OR TS=(clinical trials as topic)) OR TS=(randomly)) OR TI=(trial)		2,027,201
#9	(TS=(animals)) NOT TS=(humans)		1,173,867
#10	(#8) NOT #9		1,961,418
#11	#7 AND #10		1967
#12	#11 AND DT=(Article)		1682

Table S5. Summary of included research outcomes.

Outcomes	Number of studies	Reference number in Table 1
**BMD**		
Lumbar spine (LS) BMD	32	2, 3, 6, 7, 10, 12, 13, 14, 16, 18, 20, 22, 23, 24, 25, 29, 30, 32, 34, 35, 36, 37, 43, 46, 47, 49, 51, 52, 55, 56, 58, 60
Total body (TB) BMD	20	3, 6, 10, 12, 13, 14, 16, 23, 24, 29, 30, 35, 39, 40, 47, 52, 54, 55, 56, 60
Femoral neck (FN) BMD	24	3, 7, 12, 13, 14, 16, 18, 20, 22, 23, 24, 29, 30, 32, 36, 37, 42, 43, 47, 52, 55, 56, 58, 59
Total hip (TH) BMD	16	6, 12, 13, 14, 16, 20, 25, 29, 30, 32, 47, 54, 55, 56, 58, 59
Trochanter BMD	13	3, 13, 14, 16, 18, 21, 24, 25, 42, 47, 55, 56, 58
Intertrochanter BMD	10	3, 13, 21, 25, 29, 30, 42, 47, 56, 58
Ward triangle (WT) BMD	10	3, 14, 16, 22, 24, 25, 37, 47, 55, 56
Total spine (TS) BMD	4	21, 35, 40, 54
Whole femurs (WF) BMD	3	23, 24, 52
Arms BMD	2	35, 40
Femoral trochanter BMD	2	22, 52
Legs BMD	2	35, 40
Pelvis BMD	2	35, 40
Additional forearm sites BMD (radius 1/3 of styloid process and whole forearms)	1	23
Additional forearm sites BMD (forearm 33% radius, ultradistal radius, and total radius)	1	24
Additional forearm sites BMD (Dist R+U 1/10)	1	60
**Bone turnover markers**		
Osteocalcin (OC)	23	1, 4, 6, 8, 12, 18, 21, 23, 26, 27, 31, 33, 35, 37, 38, 45, 48, 49, 50, 53, 54, 55, 58
Deoxypyridinoline (DPD)	18	5, 6, 10, 11, 12, 15, 17, 19, 22, 31, 36, 37, 38, 50, 53, 55, 57, 58
25-hydroxyvitamin D [25(OH)D]	14	8, 9, 10, 12, 23, 26, 28, 32, 33, 35, 36, 37, 45, 54
C-terminal telopeptide of type I collagen (CTx)	16	1, 8, 19, 23, 26, 27, 28, 31, 33, 35, 41, 44, 48, 49, 53, 54
N-terminal telopeptide of type I collagen (NTx)	12	4, 18, 19, 22, 23, 24, 31, 32, 45, 46, 49, 54
Bone-specific alkaline phosphatase (BAP)	19	5, 6, 8, 10, 11, 21, 22, 24, 25, 31, 33, 36, 37, 45, 46, 53, 54, 55, 58
Parathyroid hormone (PTH)	12	8, 10, 12, 19, 23, 26, 27, 28, 35, 37, 45, 54
Procollagen type I N-terminal propeptide (PINP)	10	8, 10, 19, 26, 27, 28, 33, 41, 44, 54
Insulin-like growth factor 1 (IGF-1)	8	5, 6, 8, 26, 35, 36, 53, 59
Pyridinoline (Pyr)	6	10, 17, 36, 50, 51, 57

Table S6. Inconsistency test between direct and indirect treatment comparisons in mixed treatment comparison for LS BMD.

Side	Number of studies	Direct		Indirect		Difference		P>z	tau
		Coef.	SE	Coef.	SE	Coef.	SE		
Comparison of dairy and soy products									
CON vs MK	2	.	.	.	.	.	.	.	0.0395
CON vs MK + YOG	2	.	.	.	.	.	.	.	.
CON vs MP	3	.	.	.	.	.	.	.	.
CON vs SMK	1	-2.48e-13	0.0545	-0.156	0.148	0.156	0.158	0.322	0.0501
MK vs SMK	1	-0.35	0.0539	0.121	0.148	-0.156	0.158	0.322	0.0501
Comparison of milk protein, soy protein, and soy isoflavones									
CON vs SP	1	0.0882	0.0349	-0.0003	0.0313	0.0886	0.0468	0.059	0.0322
CON vs ISO	17	0.0221	0.0108	0.0510	0.0652	-0.0290	0.0661	0.662	0.0348
CON vs SP + ISO	2	0.114	0.241	0.0390	0.0444	-0.0276	0.0506	0.586	0.0347
CON vs MBP	3	.	.	.	.	.	.	.	.
SP vs ISO	2	-0.253	0.318	0.00230	0.0396	-0.0276	0.0506	0.586	0.0347
SP vs SP + ISO	3	-0.0236	0.248	0.00538	0.0617	-0.0290	0.0662	0.661	0.0348
SP vs WP	1	0.0560	0.0501	-0.1126	0.0961	0.169	0.106	0.111	0.0322
SP vs CAS + WP	1	0.0140	0.445	-0.0450	0.0938	0.0590	0.104	0.570	0.0344
ISO vs SP + ISO	1	-0.0301	0.0362	0.00092	0.0275	-0.0393	0.0456	0.389	0.0344
SP + ISO vs CAS	1	0.0200	0.0585	-0.0347	13.170	0.0547	13.170	0.997	0.0341
SP + ISO vs WP	1	0.0110	0.0487	0.180	0.0972	-0.169	0.106	0.111	0.0322
SP + ISO vs CAS + WP	1	0.0120	0.0445	0.0710	0.0938	-0.0590	0.104	0.570	0.0344

SE, standard error; CON: control (placebo/no treatment); SP: soy protein; ISO: isoflavone; SP + ISO: soy protein + isoflavone; MBP: milk basic protein; CAS: casein; WP: whey protein; CAS + WP: casein + whey protein; MK: milk; MK + YOG: milk + yogurt; MP: milk powder; SMK: soymilk.

Table S7. Inconsistency test between direct and indirect treatment comparisons in mixed treatment comparison for TB BMD.

Side	Number of studies	Direct		Indirect		Difference		P>z	tau
		Coef.	SE	Coef.	SE	Coef.	SE		
Comparison of dairy and soy products									
CON vs MK	1	.	.	.	.	.	.	.	.
CON vs MK + YOG	3	.	.	.	.	.	.	.	.
CON vs MP	2	.	.	.	.	.	.	.	.
Comparison of milk protein, soy protein, and soy isoflavones									
CON vs SP	1	0.0367	0.00679	0.0116	0.0155	0.0251	0.0169	0.137	0.00463
CON vs ISO	7	-0.00275	0.00479	0.0287	0.0435	-0.0315	0.0436	0.471	0.00449
CON vs SP + ISO	2	0.00791	0.00934	0.0134	0.0170	-0.00554	0.0194	0.775	0.00814
CON vs MBP	1	.	.	.	.	.	.	.	.
SP vs ISO	2	-0.0325	0.00974	-0.0269	0.0181	-0.00554	0.0194	0.775	0.00814
SP vs SP + ISO	2	-0.0228	0.00870	0.00870	0.0433	-0.0315	0.0436	0.470	0.00449
SP vs CAS + WP	1	0.0160	0.01410	-0.0375	0.0263	0.0535	0.0299	0.073	4.50e-10
ISO vs SP + ISO	1	0.00938	0.00677	0.0209	0.0142	-0.0115	0.0154	0.454	0.00489
SP + ISO vs CAS + WP	1	0.0160	0.0142	0.0695	0.0262	-0.0535	0.0299	0.073	4.673e-10

SE, standard error; CON: control (placebo/no treatment); SP: soy protein; ISO: isoflavone; SP + ISO: soy protein + isoflavone; MBP: milk basic protein; CAS + WP: casein + whey protein; MK: milk; MK + YOG: milk + yogurt; MP: milk powder.

Table S8. Inconsistency test between direct and indirect treatment comparisons in mixed treatment comparison for OC.

Side	Number of studies	Direct		Indirect		Difference		P>z	tau
		Coef.	SE	Coef.	SE	Coef.	SE		
Comparison of dairy and soy products									
CON vs MK	1	.	.	.	.	.	.	.	.
CON vs MK + YOG	1	.	.	.	.	.	.	.	.
CON vs MP	4	.	.	.	.	.	.	.	.
Comparison of milk protein, soy protein, and soy isoflavones									
CON vs ISO	11	-0.146	0.717	6.11	6.27	-6.25	6.31	0.322	2.17
CON vs SP + ISO	1	1.9	3.68	-4.35	5.13	6.25	6.31	0.322	2.17
CON vs MBP	1	.	.	.	.	.	.	.	.
SP vs ISO	1	3.51	4.30	-2.74	4.63	6.25	6.31	0.322	2.17
SP vs CAS + WP	1	-0.700	2.71	5.56	5.70	-6.26	6.31	0.322	2.17
SP vs CAS + WP	1	-2.10	2.59	10.4	12.4	-12.5	12.6	0.322	2.17
SP + ISO vs CAS + WP	1	-1.40	2.70	-13.9	12.3	12.5	12.6	0.322	2.17

SE, standard error; CON: control (placebo/no treatment); SP: soy protein; ISO: isoflavone; SP + ISO: soy protein + isoflavone; MBP: milk basic protein; CAS + WP: casein + whey protein; MK: milk; MK + YOG: milk + yogurt; MP: milk powder.

Table S9. Inconsistency test between direct and indirect treatment comparisons in mixed treatment comparison for DPD.

Side	Number of studies	Direct		Indirect		Difference		P>z	tau
		Coef.	SE	Coef.	SE	Coef.	SE		
Comparison of dairy and soy products									
CON vs MK	1	.	.	.	.	.	.	.	.
CON vs MP	2	.	.	.	.	.	.	.	.
Comparison of milk protein, soy protein, and soy isoflavones									
CON vs ISO	10	.	.	.	.	.	.	.	.
CON vs SP + ISO	3	.	.	.	.	.	.	.	.

SE, standard error; CON: control (placebo/no treatment); ISO: isoflavone; SP + ISO: soy protein + isoflavone; MK: milk; MP: milk powder.

Table S10. Inconsistency test between direct and indirect treatment comparisons in mixed treatment comparison for CTx.

Side	Number of studies	Direct		Indirect		Difference		P>z	tau
		Coef.	SE	Coef.	SE	Coef.	SE		
Comparison of dairy and soy products									
CON vs MK	1	.	.	.	.	.	.	.	.
CON vs MK + YOG	1	.	.	.	.	.	.	.	.
CON vs MP	4	.	.	.	.	.	.	.	.
Comparison of milk protein, soy protein, and soy isoflavones									
CON vs ISO	3	.	.	.	.	.	.	.	.
CON vs CAS + WP	1	.	.	.	.	.	.	.	.

SE, standard error; CON: control (placebo/no treatment); SP: soy protein; ISO: isoflavone; SP + ISO: soy protein + isoflavone; CAS + WP: casein + whey protein; MK: milk; MK +YOG: milk + yogurt; MP: milk powder.

Table S11. Inconsistency test between direct and indirect treatment comparisons in mixed treatment comparison for PINP.

Side	Number of studies	Direct		Indirect		Difference		P>z	tau
		Coef.	SE	Coef.	SE	Coef.	SE		
Comparison of dairy and soy products									
CON vs MK	2	.	.	.	.	.	.	.	.
CON vs MP	4	.	.	.	.	.	.	.	.

SE, standard error; CON: control (placebo/no treatment); SP: soy protein; ISO: isoflavone; SP + ISO: soy protein + isoflavone; CAS + WP: casein + whey protein; MK: milk; MP: milk powder.

Table S12. Loop-specific heterogeneity for LS BMD.

Loop	IF	seIF	z_value	p_value	CI_95	Loop_Heterog_tau2
Comparison of dairy and soy products						
CON-MK-SMK	0.078	0.041	1.882	0.060	(0.00,0.16)	0.000
Comparison of milk protein, soy protein, and soy isoflavones						
SP- SP + ISO-WP	0.114	0.054	2.111	0.035	(0.01,0.22)	0.000
CON-SP-SP + ISO	0.093	0.028	3.318	0.001	(0.04,0.15)	0.000
SP-ISO-SP + ISO	0.070	0.041	1.683	0.092	(0.00,0.15)	0.000
SP-SP + ISO-CAS + WP	0.057	0.082	0.695	0.487	(0.00,0.22)	0.000
CON-SP-ISO	0.047	0.059	0.789	0.430	(0.00,0.16)	0.002
CON-ISO-SP + ISO	0.021	0.050	0.411	0.681	(0.00,0.12)	0.002

CON: control (placebo/no treatment); SP: soy protein; ISO: isoflavone; SP + ISO: soy protein + isoflavone; MBP: milk basic protein; CAS: casein; WP: whey protein; CAS + WP: casein + whey protein; MK: milk; MK + YOG: milk + yogurt; MP: milk powder; SMK: soymilk.

Table S13. Loop-specific heterogeneity for TB BMD.

Loop	IF	seIF	z_value	p_value	CI_95	Loop_Heterog_tau2
Comparison of milk protein, soy protein, and soy isoflavones						
SP-ISO-SP + ISO	0.027	0.021	1.314	0.189	(0.00, 0.07)	0.000
CON-SP-SP + ISO	0.026	0.015	1.702	0.089	(0.00, 0.06)	0.000
SP-SP + ISO-CAS + WP	0.012	0.028	0.422	0.673	(0.00, 0.07)	0.000
CON-SP-ISO	0.007	0.011	0.588	0.557	(0.00, 0.03)	0.000
CON-ISO-SP + ISO	0.005	0.009	0.496	0.620	(0.00, 0.02)	0.000

CON: control (placebo/no treatment); SP: soy protein; ISO: isoflavone; SP + ISO: soy protein + isoflavone; MBP: milk basic protein; CAS + WP: casein + whey protein; MK: milk; MK + YOG: milk + yogurt; MP: milk powder.

Table S14. Loop-specific heterogeneity for OC.

Loop	IF	seIF	z_value	p_value	CI_95	Loop_Heterog_tau2
Comparison of milk protein, soy protein, and soy isoflavones						
CON-SP-ISO-SP + ISO	6.315	10.137	0.623	0.533	(0.00, 26.18)	0.274
SP-SP + ISO-CAS + WP	.	.	.	.	.	0.000

CON: control (placebo/no treatment); SP: soy protein; ISO: isoflavone; SP + ISO: soy protein + isoflavone; MBP: milk basic protein; CAS + WP: casein + whey protein; MK: milk; MK + YOG: milk + yogurt; MP: milk powder.

Table S15. Comparative results of network meta-analysis of bone turnover markers CTx and PINP of the included studies.

**(a) Comparison of dairy and soy products**				**(b) Comparison of milk protein, soy protein, and soy isoflavones**		
**CON**	0.03<br>(-0.24, 0.30)	-0.13<br>(-0.51,0.25)	-0.02<br>(-0.33,0.28)	-0.11<br>(-0.44,0.22)	-0.06<br>(-0.23,0.11)	**CON**
**CTx**	**MK + YOG**	-0.10<br>(-0.37,0.17)	-0.15<br>(-0.45,0.14)	-0.17<br>(-0.45,0.11)	**CAS + WP**	**CTx**
**PINP**	**CTx**	**MK**	-0.12<br>(-0.26,0.01)	**ISO**	**CTx**	**PINP**
**MK**	**PINP**	**CTx**	**MP**	**CTx**	**PINP**	-
-2.01<br>(-19.76,15.73)	**MP**	**PINP**	**CTx**	**PINP**	-	-
-3.79<br>(-29.85,22.27)	-3.32<br>(-13.17,6.54)	**CON**	**PINP**	-	-	-

CON: control (placebo/no treatment); MK: milk; MK + YOG: milk + yogurt; MP: milk powder; ISO: isoflavone; CAS + WP: casein + whey protein.

Table S16. Comparative results of subgroup analysis in LS and TB BMD.

**(a) BMI < 24.0**				**(b) BMI ≥ 24.0**						
**CON**	-0.04<br>(-0.13,0.06)	0.00<br>(-0.02,0.02)	0.02<br>(-0.00,0.04)	0.07<br>(-0.03,0.17)	0.05<br>(-0.01,0.11)	0.05<br>(-0.04,0.14)	0.04<br>(-0.08,0.17)	0.03<br>(0.00,0.06)	0.02<br>(-0.02,0.07)	**CON**
**LS BMD**	**SP**	0.04<br>(-0.05,0.13)	0.06<br>(-0.04,0.15)	0.04<br>(-0.05,0.14)	0.02<br>(-0.03,0.08)	0.03<br>(-0.06,0.11)	0.02<br>(-0.10,0.14)	0.01<br>(-0.04,0.06)	**SP + ISO**	**LS BMD**
	**LS BMD**	**ISO**	0.02<br>(-0.01,0.04)	0.04<br>(-0.07,0.14)	0.02<br>(-0.04,0.08)	0.02<br>(-0.08,0.11)	0.01<br>(-0.12,0.14)	**ISO**	**LS BMD**	
		**LS BMD**	**MBP**	0.02<br>(-0.13,0.18)	0.00<br>(-0.13,0.14)	0.01<br>(-0.14,0.15)	**CAS**	**LS BMD**		**TB BMD**
**TB BMD**			**LS BMD**	0.02<br>(-0.10,0.14)	-0.00<br>(-0.09,0.08)	**CAS + WP**	**LS BMD**		**TB BMD**	**CAS + WP**
**MBP**	**TB BMD**			0.02<br>(-0.08,0.12)	**SP**	**LS BMD**		**TB BMD**	**SP**	0.00<br>(-0.02,0.03)
0.03<br>(-0.02,0.07)	**ISO**	**TB BMD**		**WP**	**LS BMD**		**TB BMD**	**SP + ISO**	0.02<br>(0.01,0.04)	0.03<br>(0.00,0.05)
0.05<br>(-0.04,0.13)	0.02<br>(-0.05,0.09)	**SP**	**TB BMD**	**LS BMD**		**TB BMD**	**ISO**	0.01<br>(-0.00,0.03)	**0.04**<br>**(0.02,0.05)**	**0.04**<br>**(0.01,0.07)**
0.02<br>(-0.02,0.07)	-0.00<br>(-0.02,0.02)	-0.02 (-<br>0.09,0.05)	**CON**		**TB BMD**	**CON**	-0.00 (-<br>0.01,0.01)	0.01 (-<br>0.00,0.02)	**0.03**<br>**(0.02,0.05)**	**0.04**<br>**(0.01,0.06)**

CON: control (placebo/no treatment); SP: soy protein; ISO: isoflavone; SP + ISO: soy protein + isoflavone; MBP: milk basic protein; CAS: casein; WP: whey protein; CAS + WP: casein + whey protein

Table S17. Comparative results of subgroup analysis in bone turnover markers OC and DPD.

(a) BMI < 24.0

**CON**	-1.71<br>(-3.59,0.17)	0.10<br>(-0.51,0.72)
**OC**	**MBP**	1.81<br>(-0.16,3.79)
	**OC**	**ISO**
**DPD**		**OC**
**ISO**	**DPD**	
0.66<br>(-0.73,2.04)	**CON**	**DPD**

(b) BMI ≥ 24.0

2.22<br>(-8.06,12.51)	0.83<br>(-7.62,9.29)	0.07<br>(-7.92,8.05)	-0.32<br>(-3.91,3.28)	**CON**
2.54<br>(-7.79,12.87)	1.15<br>(-7.18,9.48)	0.38<br>(-7.85,8.62)	**ISO**	**OC**
2.16<br>(-5.94,10.26)	0.77<br>(-6.54,8.08)	**SP + ISO**	**OC**	**DPD**
1.39<br>(-6.61,9.39)	**SP**	**OC**	**DPD**	**ISO**
**CAS + WP**	**OC**	**DPD**	**SP + ISO**	1.23<br>(-2.26,4.72)
**OC**	**DPD**	**CON**	0.41<br>(-2.24,3.07)	1.64<br>(-0.62,3.91)

CON: control (placebo/no treatment); SP: soy protein; ISO: isoflavone; SP + ISO: soy protein + isoflavone; MBP: milk basic protein; CAS + WP: casein + whey protein

Table S18. Subgroup analysis of intervention rankings using surface under the cumulative ranking (SUCRA) values.

Rank	LS BMD		TB BMD		OC		DPD	
	**Intervention**	**SUCRA (%)**	**Intervention**	**SUCRA (%)**	**Intervention**	**SUCRA (%)**	**Intervention**	**SUCRA (%)**
BMI < 24.0								
1	MBP	90.3	MBP	85.3	ISO	79.6	CON	82.1
2	ISO	50.3	CON	48.7	CON	66.8	ISO	17.9
3	CON	41.5	ISO	42.8	MBP	3.6		
4	SP	17.9	SP	23.2				
BMI ⩾ 24.0								
1	WP	72.5	CAS + WP	90.3	CAS + WP	66.6	ISO	86.3
2	SP	65.0	SP	84.0	SP	53.4	SP + ISO	43.2
3	CAS + WP	61.3	SP + ISO	47.7	CON	45.8	CON	23.2
4	CAS	54.8	CON	19.1	SP + ISO	44.0		
5	ISO	47.6	ISO	8.8	ISO	40.3		
6	SP + ISO	37.3						
7	CON	11.4						

CON: control (placebo/no treatment); SP: soy protein; ISO: isoflavone; SP + ISO: soy protein + isoflavone; MBP: milk basic protein; CAS: casein; WP: whey protein; CAS + WP: casein + whey protein

Table S19. Comparative results of sensitivity analysis in LS and TB BMD (excluding MBP).

**Comparison of milk protein, soy protein, and soy isoflavones**						
0.06	0.04	0.04	0.04	0.02	0.02	**CON**
(-0.04,0.16)	(-0.01,0.09)	(-0.05,0.13)	(-0.09,0.16)	(0.00,0.04)	(-0.02,0.06)	
0.04	0.02	0.02	0.02	0.01	**SP + ISO**	**LS BMD**
(-0.05,0.13)	(-0.03,0.07)	(-0.06,0.10)	(-0.10,0.14)	(-0.04,0.05)		
0.04	0.01	0.02	0.01	**ISO**	**LS BMD**	
(-0.06,0.13)	(-0.04,0.06)	(-0.07,0.10)	(-0.11,0.14)			**TB BMD**
0.02	-0.00	0.00	**CAS**	**LS BMD**		
(-0.13,0.17)	(-0.13,0.12)	(-0.14,0.14)			**TB BMD**	**CAS + WP**
0.02	-0.00	**CAS + WP**	**LS BMD**			0.01
(-0.10,0.14)	(-0.08,0.08)			**TB BMD**	**SP**	(-0.02,0.03)
0.02	**SP**	**LS BMD**			0.02	0.03
(-0.07,0.11)			**TB BMD**	**SP + ISO**	(0.00,0.04)	(0.00,0.05)
**WP**	**LS BMD**			0.01	**0.03**	**0.04**
		**TB BMD**	**ISO**	(-0.00,0.02)	**(0.02,0.05)**	**(0.01,0.07)**
**LS BMD**			-0.00	0.01	**0.03**	**0.04**
	**TB BMD**	**CON**	(-0.01,0.01)	(-0.00,0.02)	**(0.01,0.05)**	**(0.01,0.06)**

CON: control (placebo/no treatment); SP: soy protein; ISO: isoflavone; SP + ISO: soy protein + isoflavone; CAS: casein; WP: whey protein; CAS + WP: casein + whey protein.

Table S20. Comparative results of sensitivity analysis in bone turnover markers OC and DPD (excluding MBP).

**Comparison of milk protein, soy protein, and soy isoflavones**				
2.25 (-4.86,9.36)	0.68 (-5.51,6.87)	0.07 (-1.34,1.47)	0.23 (-5.64,6.10)	**CON**
2.02 (-3.14,7.18)	0.45 (-4.37,5.27)	-0.16 (-6.08,5.76)	**SP + ISO**	**OC**
2.18 (-4.95,9.32)	0.62 (-5.58,6.81)	**ISO**	**OC**	
1.57 (-3.41,6.55)	**SP**	**OC**		**DPD**
**CAS + WP**	**OC**		**DPD**	-
**OC**		**DPD**	-	-
	**DPD**	-	-	-

CON: control (placebo/no treatment); SP: soy protein; ISO: isoflavone; SP + ISO: soy protein + isoflavone; CAS + WP: casein + whey protein.

Table S21. Sensitivity analysis of intervention rankings using surface under the cumulative ranking (SUCRA) values (excluding MBP).

Rank	LS BMD		TB BMD		OC	
	**Intervention**	**SUCRA (%)**	**Intervention**	**SUCRA (%)**	**Intervention**	**SUCRA (%)**
Comparison of milk protein, soy protein, and soy isoflavones						
1	WP	72.6	CAS + WP	90.5	CAS + WP	74.0
2	SP	62.1	SP	83.6	SP	50.1
3	CAS + WP	60.7	SP + ISO	48.1	ISO	42.8
4	CAS	55.6	CON	19.2	SP + ISO	42.5
5	ISO	47.3	ISO	8.6	CON	40.6
6	SP + ISO	37.4				
7	CON	14.4				

CON: control (placebo/no treatment); SP: soy protein; ISO: isoflavone; SP + ISO: soy protein + isoflavone; CAS: casein; WP: whey protein; CAS + WP: casein + whey protein.

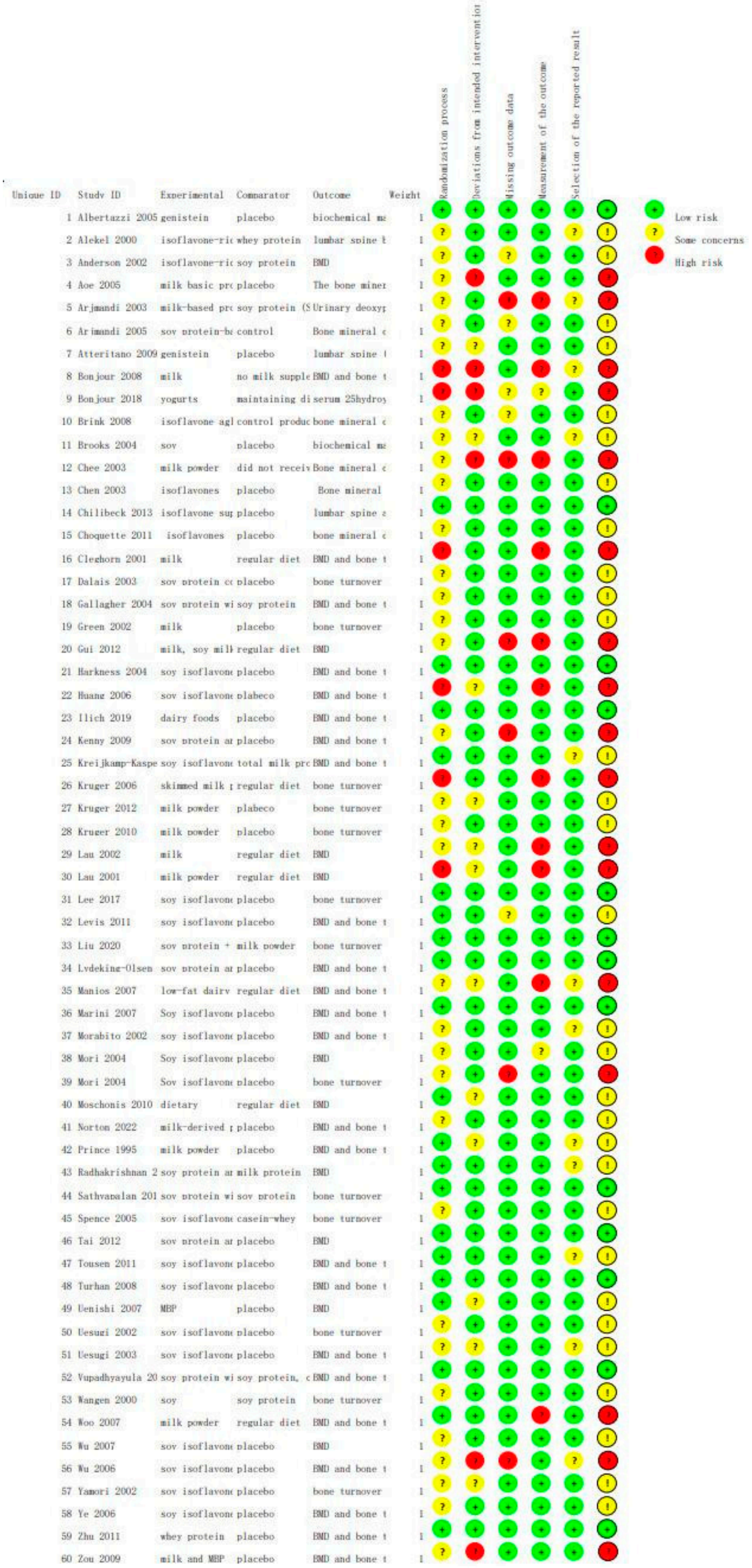

Figure S1. Graph of Cochrane risk bias assessment.

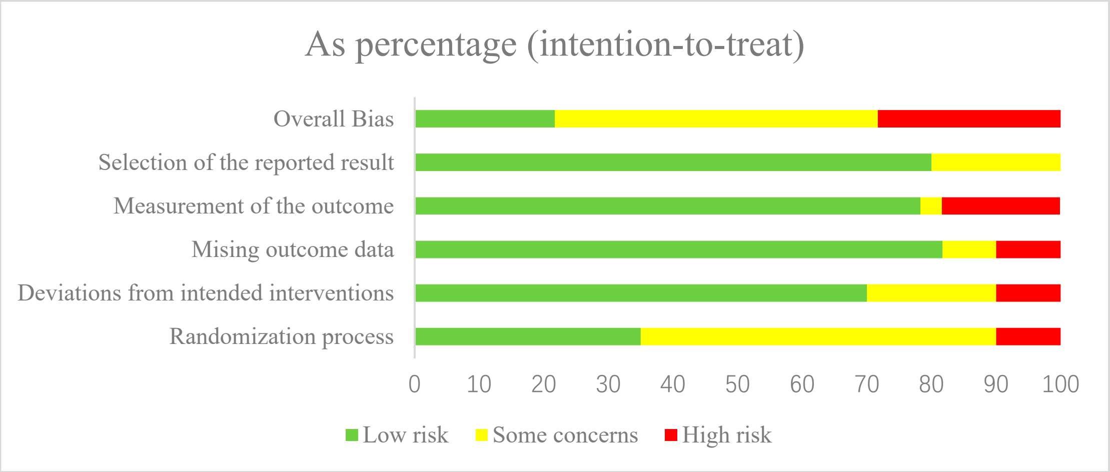

Figure S2. Bias risk of the included studies.

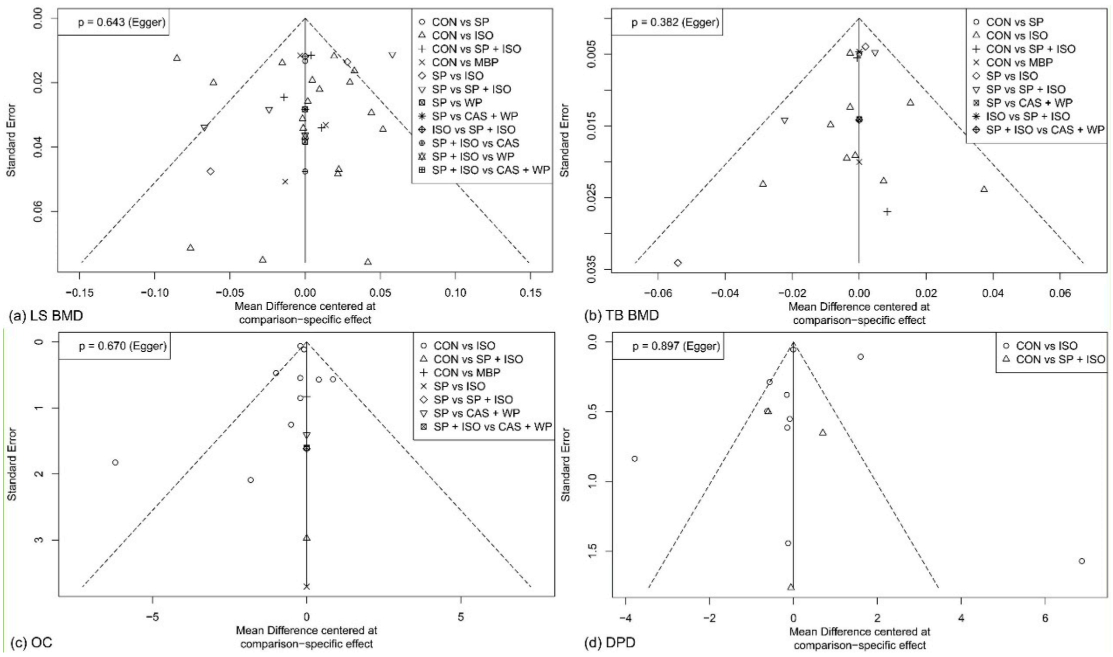

Figure S3. Publication bias assessed via funnel plots.

CON: control (placebo/no treatment); SP: soy protein; ISO: isoflavone; SP + ISO: soy protein + isoflavone; MBP: milk basic protein; CAS: casein; WP: whey protein; CAS + WP: casein + whey protein;

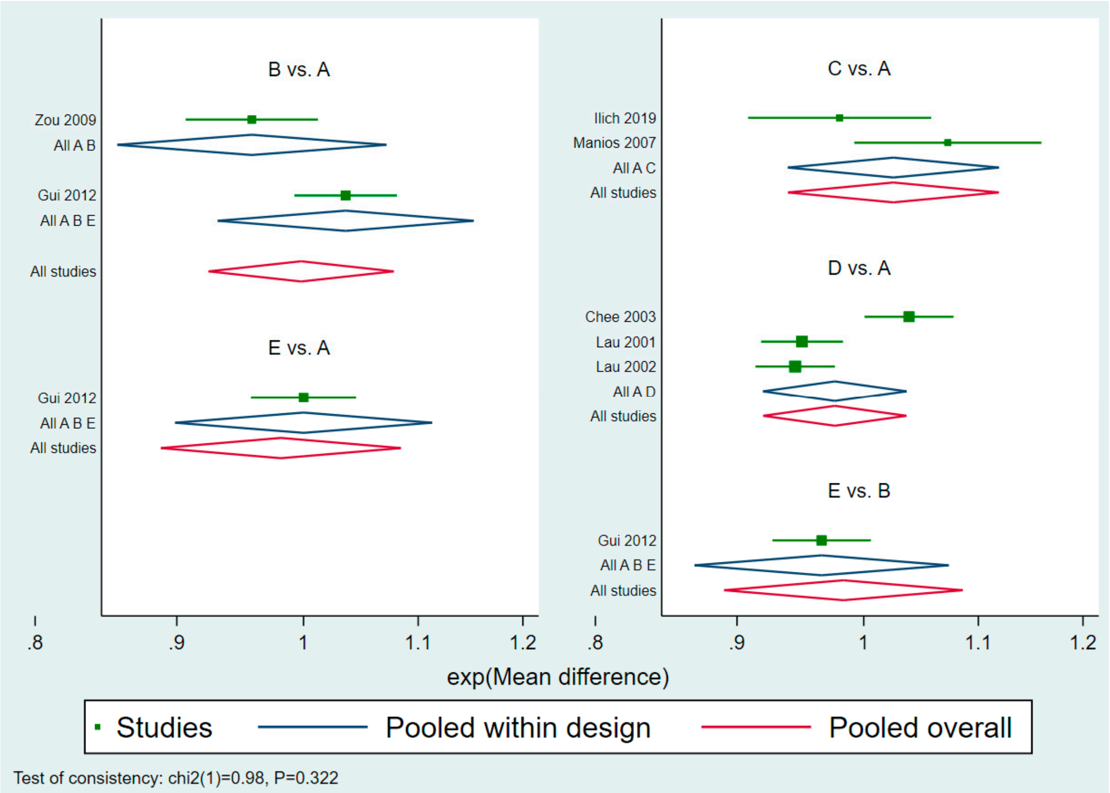

Figure S4. Global inconsistency test for LS BMD of dairy products vs. soybean.

A: placebo/no treatment; B: milk; C: milk + yogurt; D: milk powder; E: soymilk.

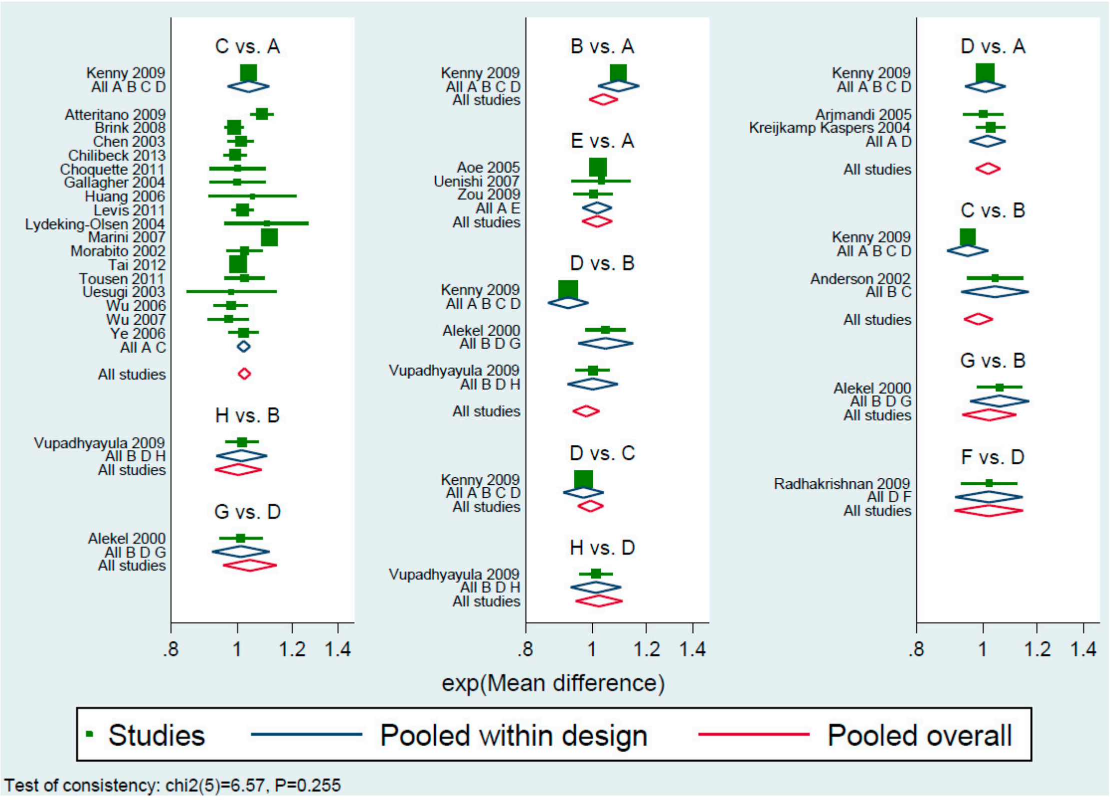

Figure S5. Global inconsistency test for LS BMD of milk-derived protein vs. soy protein and isoflavone.

A: placebo/no treatment; B: soy protein; C: isoflavone; D: soy protein + isoflavone; E: milk basic protein; F: casein; G: whey protein; H: casein + whey protein.

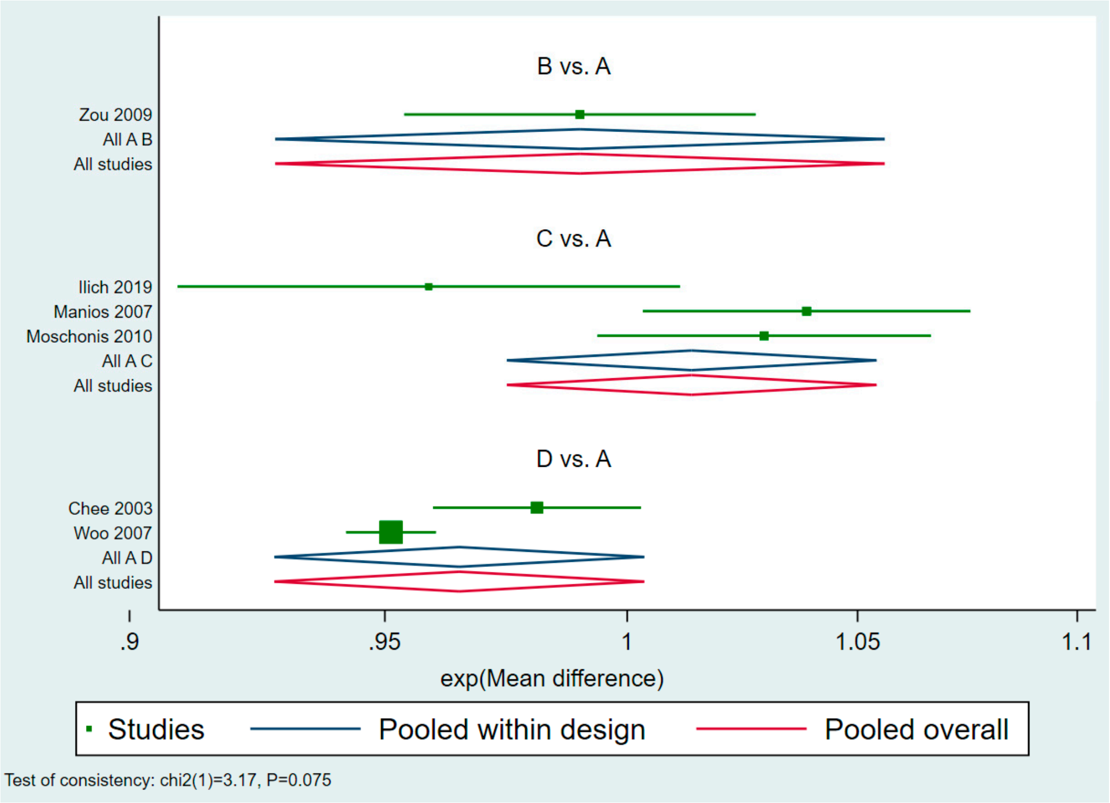

Figure S6. Global inconsistency test for TB BMD of dairy products vs. soybean.

A: placebo/no treatment; B: milk; C: milk + yogurt; D: milk powder.

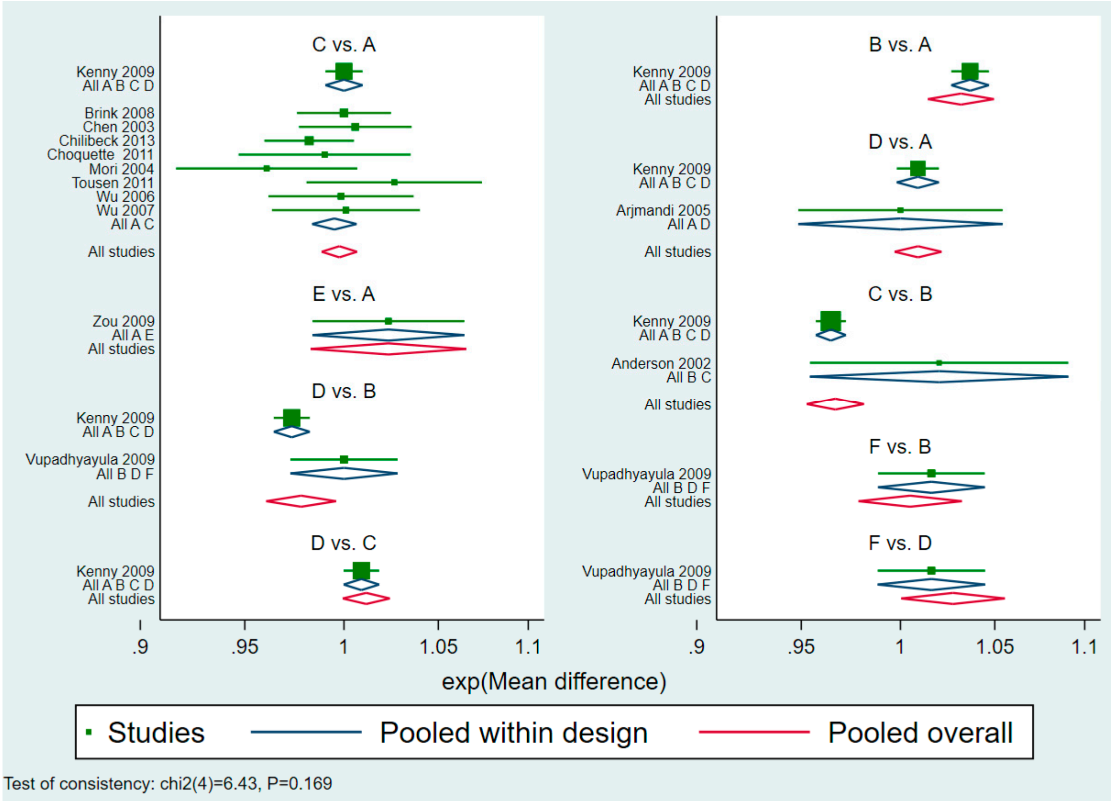

Figure S7. Global inconsistency test for TB BMD of milk-derived protein vs. soy protein and isoflavone.

A: placebo/no treatment; B: soy protein; C: isoflavone; D: soy protein + isoflavone; E: milk basic protein; F: casein + whey protein.

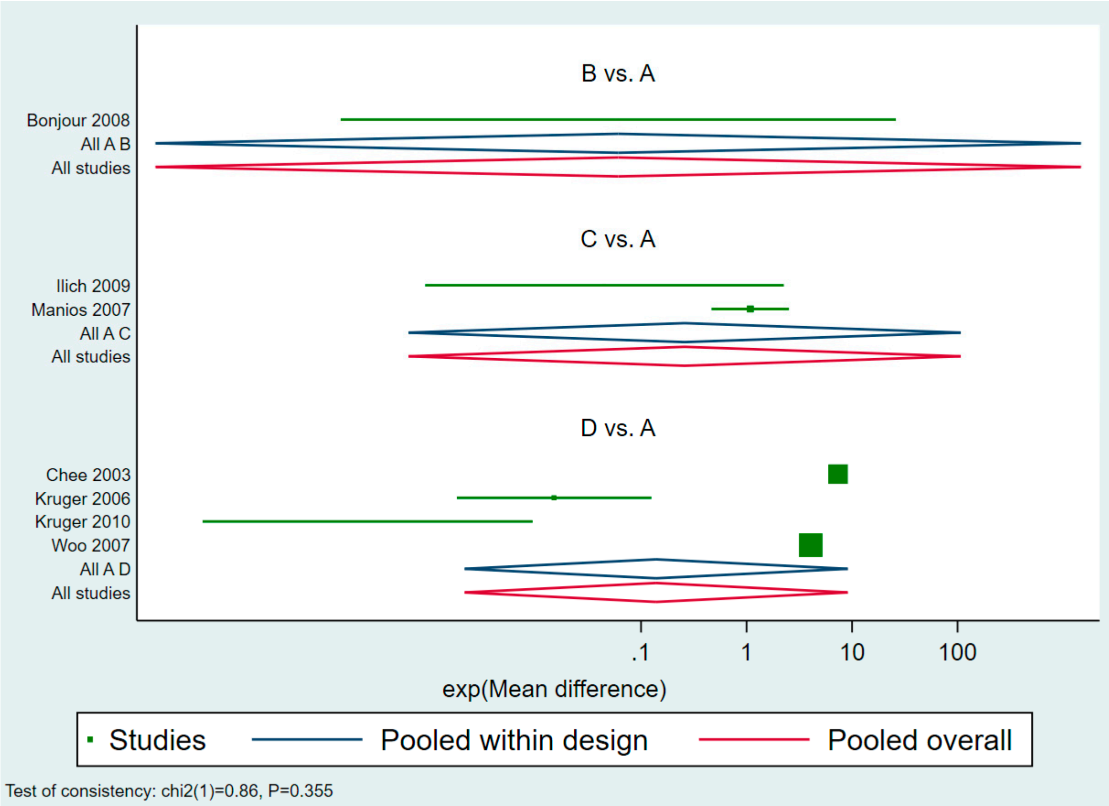

Figure S8. Global inconsistency test for OC of dairy products vs. soybean.

A: placebo/no treatment; B: milk; C: milk + yogurt; D: milk powder.

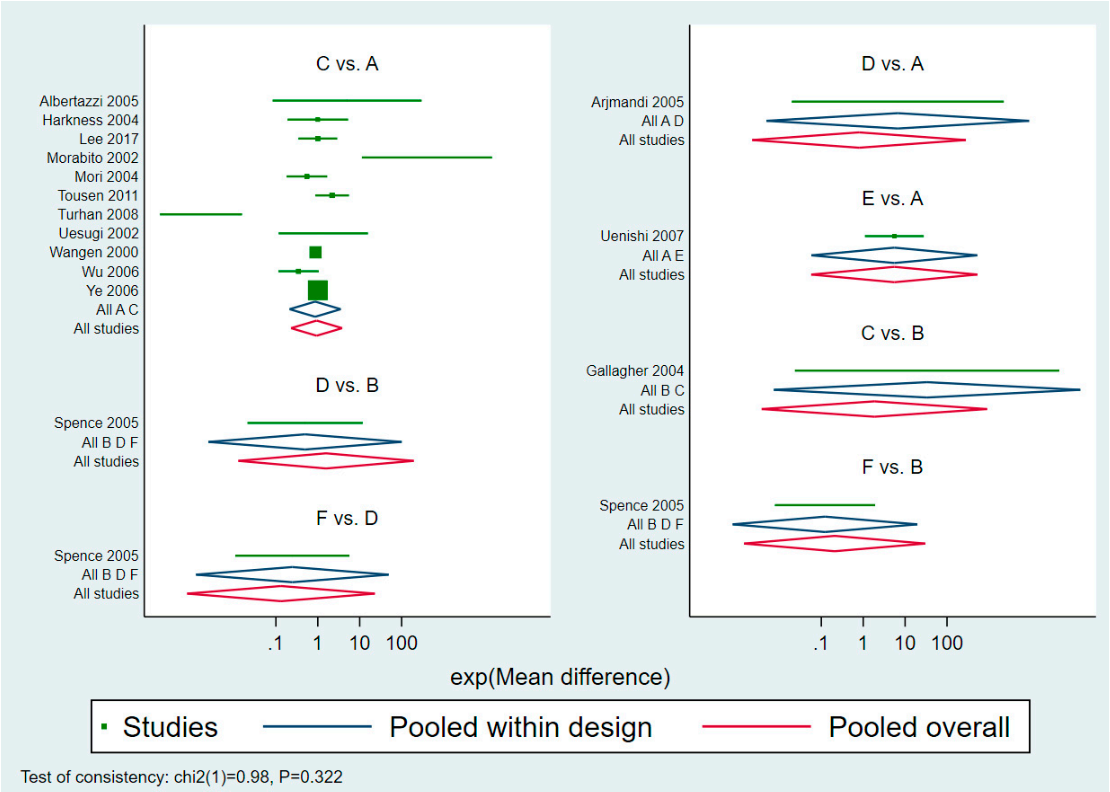

Figure S9. Global inconsistency test for OC of milk-derived protein vs. soy protein and isoflavone.

A: placebo/no treatment; B: soy protein; C: isoflavone; D: soy protein + isoflavone; E: milk basic protein; F: casein + whey protein.

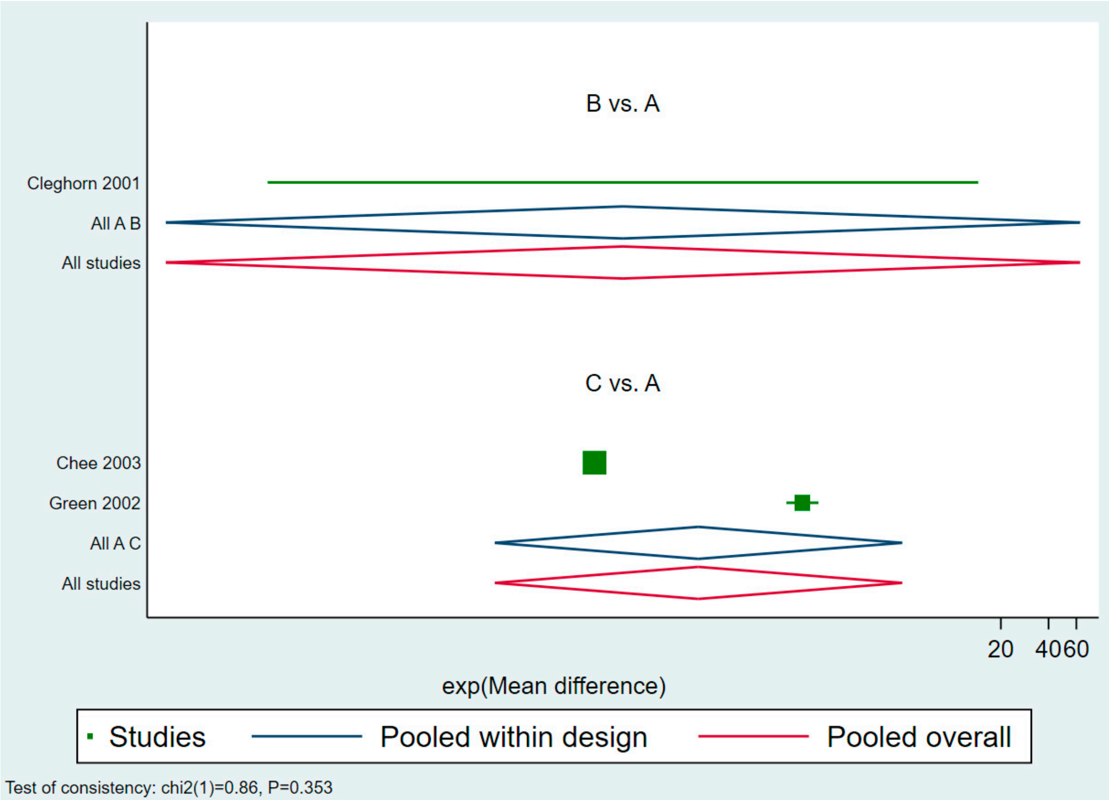

Figure S10. Global inconsistency test for DPD of dairy products vs. soybean.

A: placebo/no treatment; B: milk; C: milk powder.

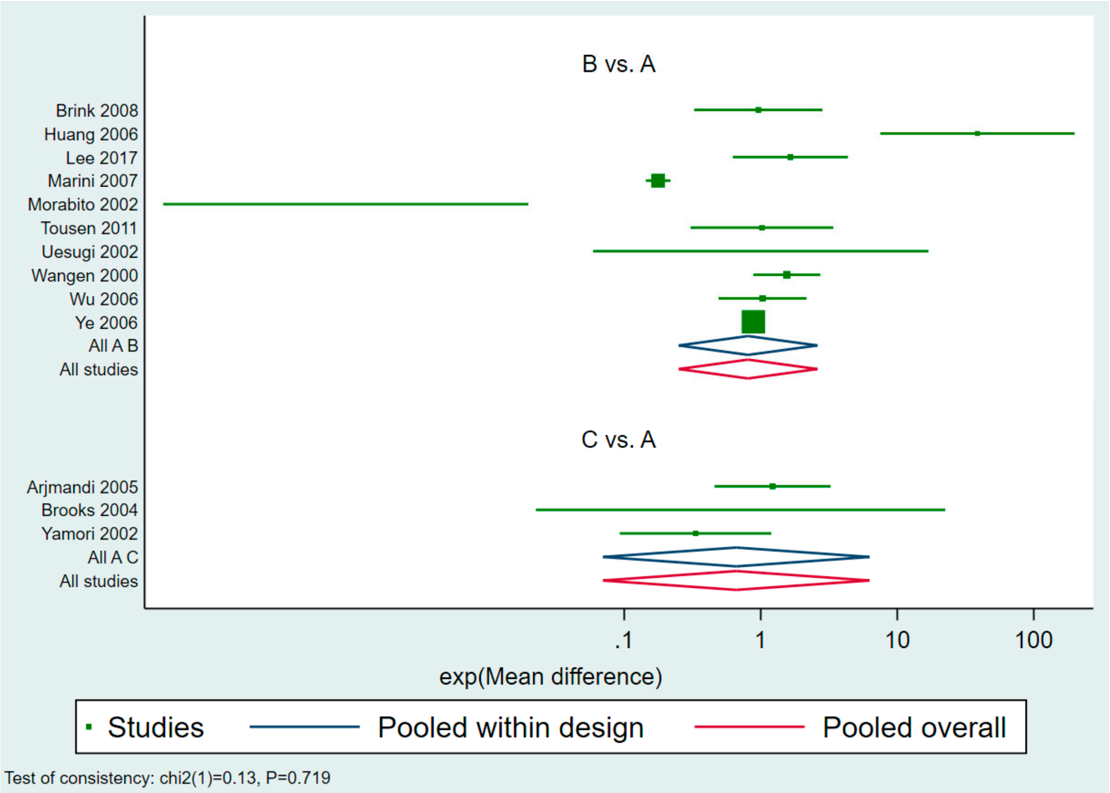

Figure S11. Global inconsistency test for DPD of milk-derived protein vs. soy protein and isoflavone.

A: placebo/no treatment; B: isoflavone; C: soy protein + isoflavone.

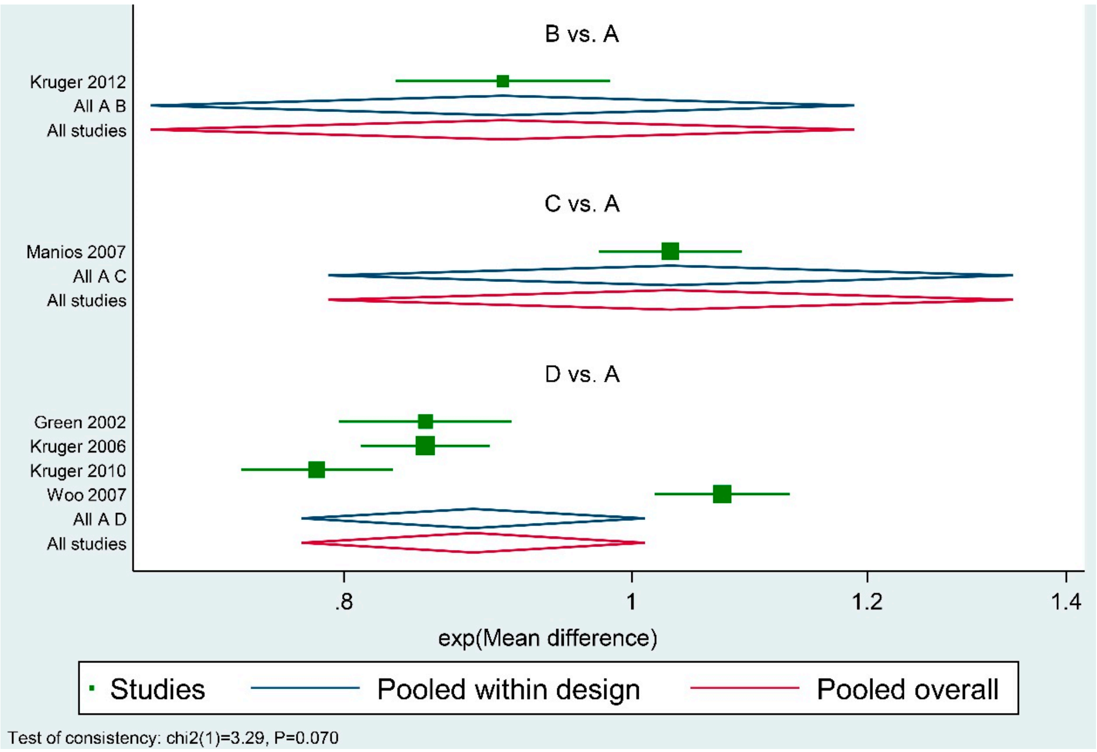

Figure S12. Global inconsistency test for CTx of dairy products vs. soybean.

A: placebo/no treatment; B: milk; C: milk + yogurt; D: milk powder.

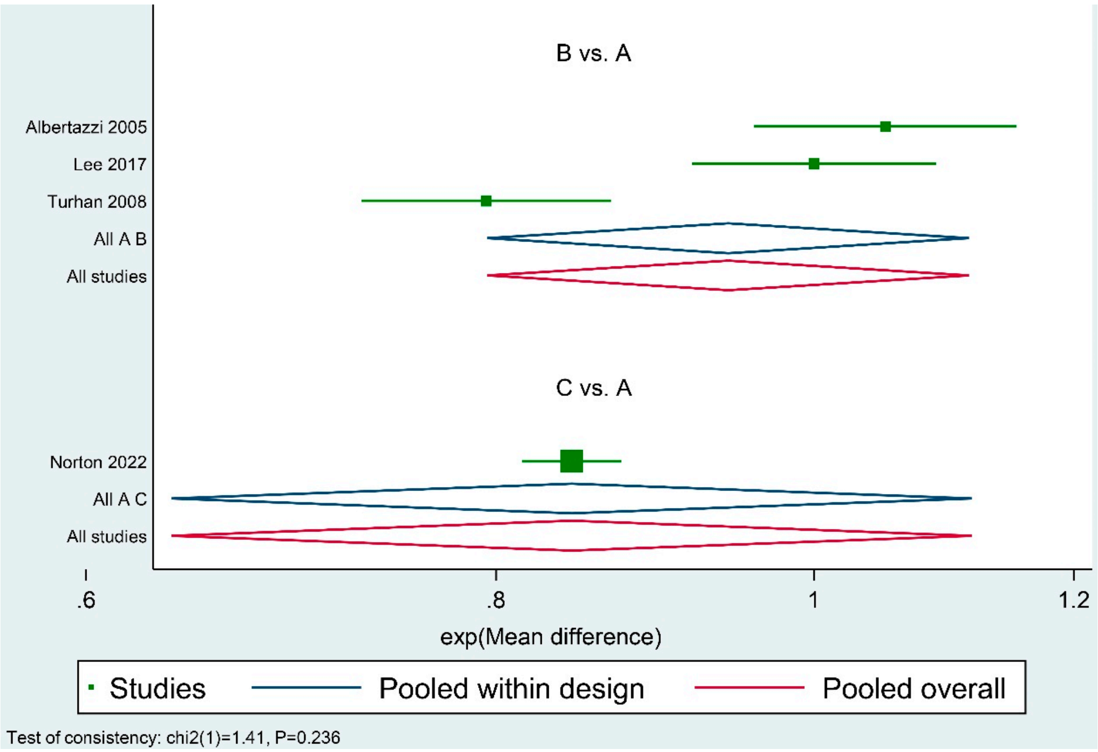

Figure S13. Global inconsistency test for CTx of milk-derived protein vs. soy protein and isoflavone.

A: placebo/no treatment; B: isoflavone; C: casein + whey protein.

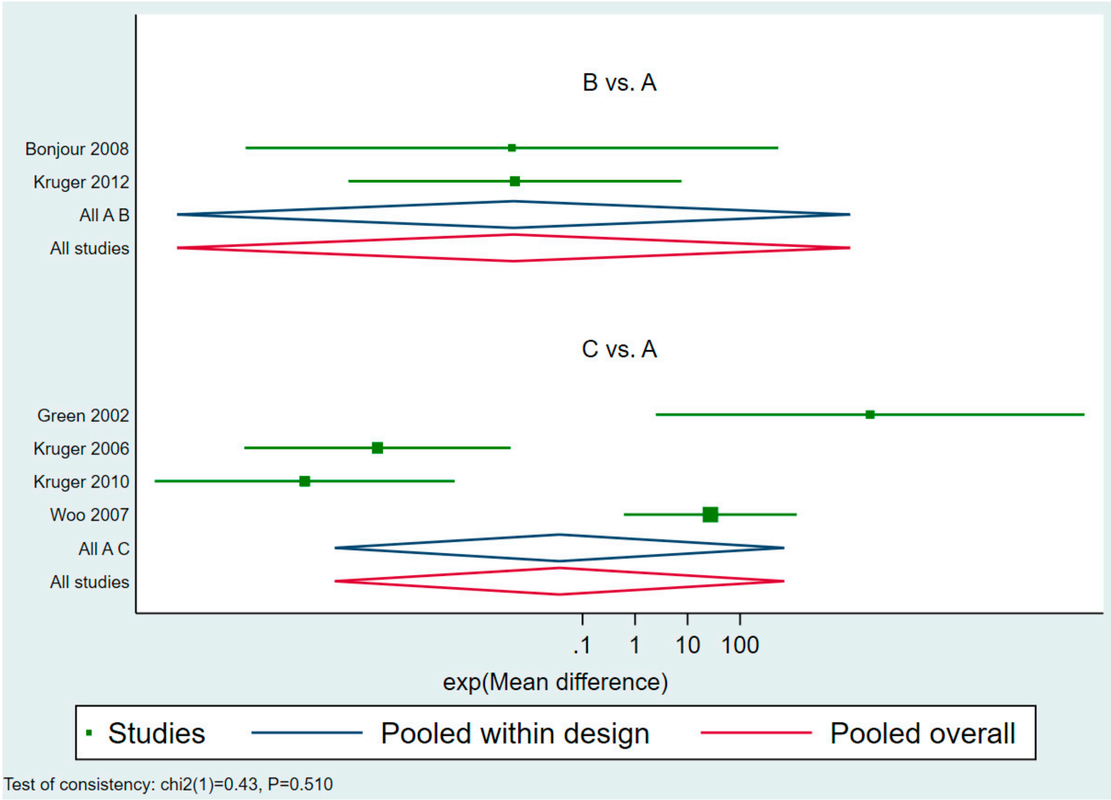

Figure S14. Global inconsistency test for PINP of dairy products vs. soybean.

A: placebo/no treatment; B: milk; C: milk powder.

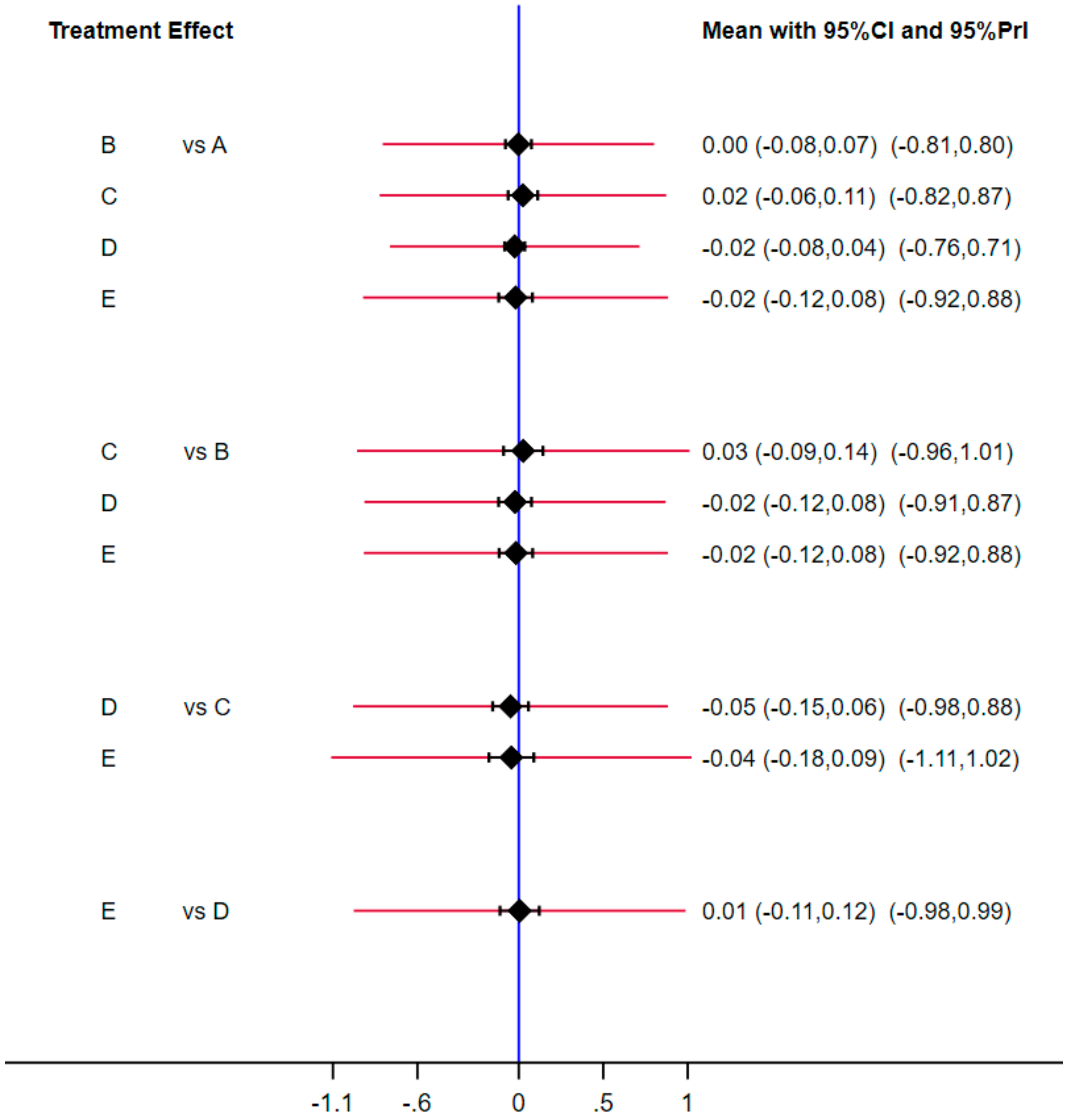

Figure S15. Effect size for LS BMD of dairy vs. soybean using forest plots.

A: placebo/no treatment; B: milk; C: milk + yogurt; D: milk powder; E: soymilk.

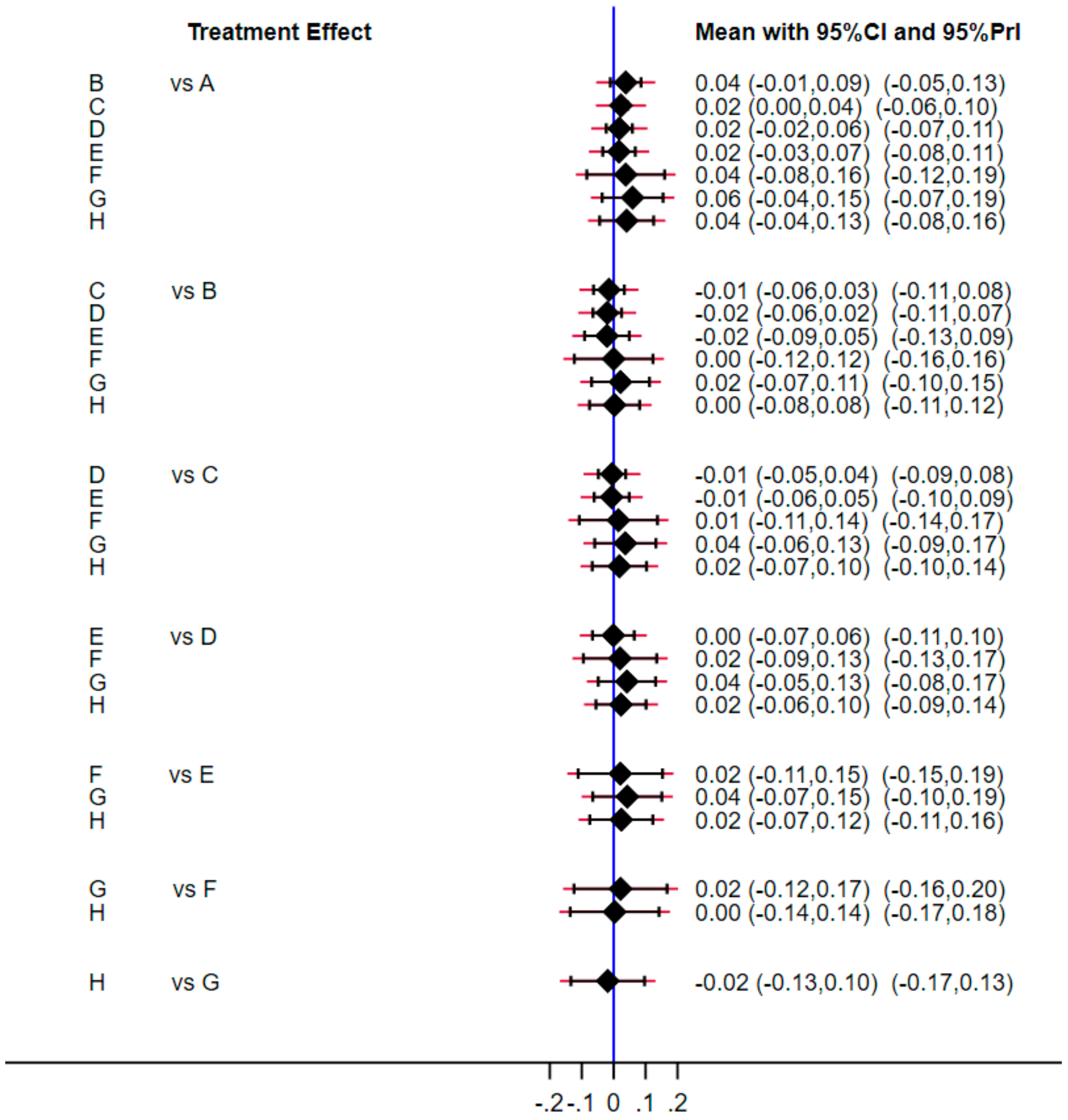

Figure S16. Effect size for LS BMD of milk-derived protein vs. soy protein and isoflavone using forest plots.

A: placebo/no treatment; B: soy protein; C: isoflavone; D: soy protein + isoflavone; E: milk basic protein; F: casein; G: whey protein; H: casein + whey protein.

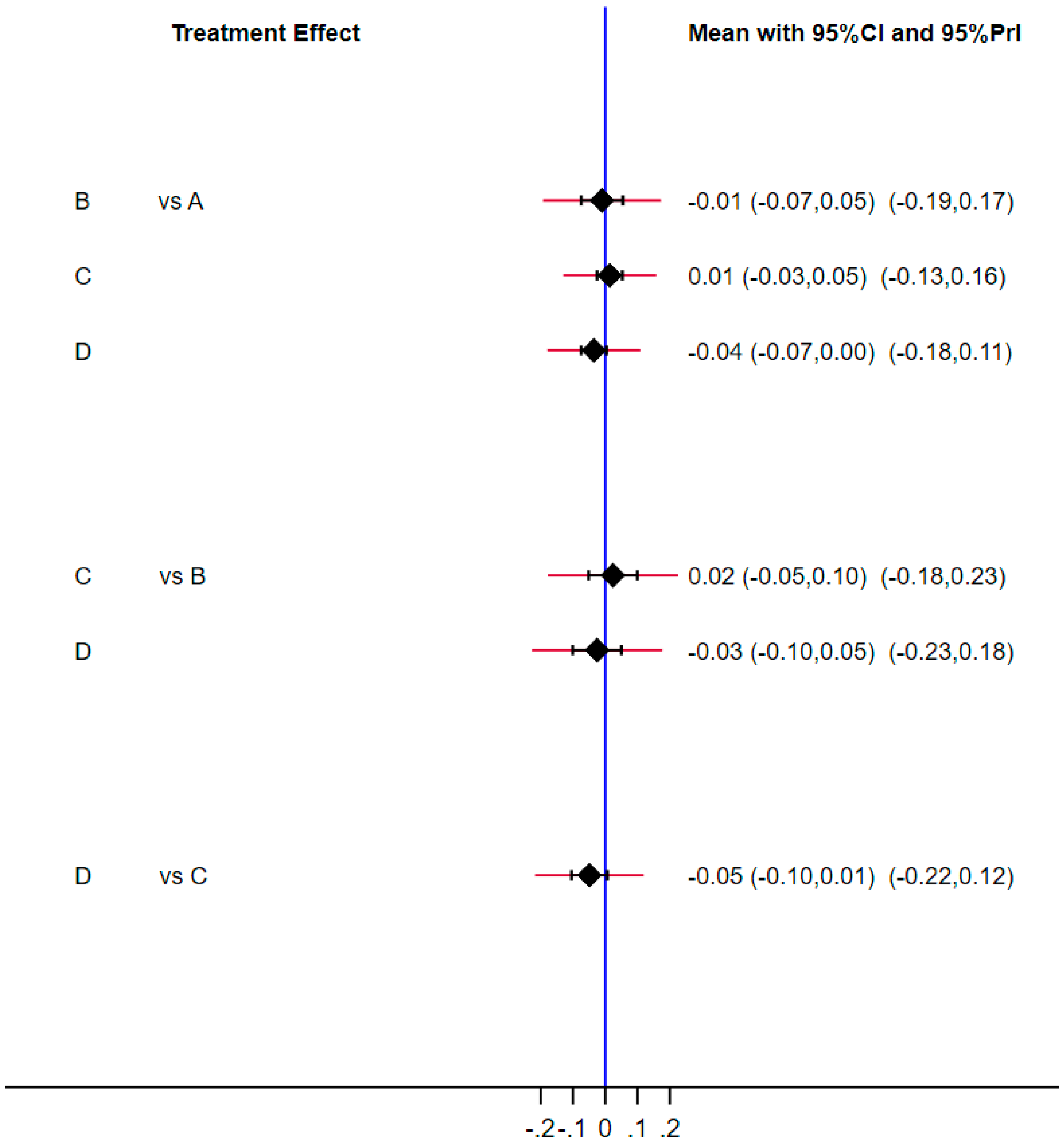

Figure S17. Effect size for TB BMD of dairy products using forest plots.

A: placebo/no treatment; B: milk; C: milk + yogurt; D: milk powder.

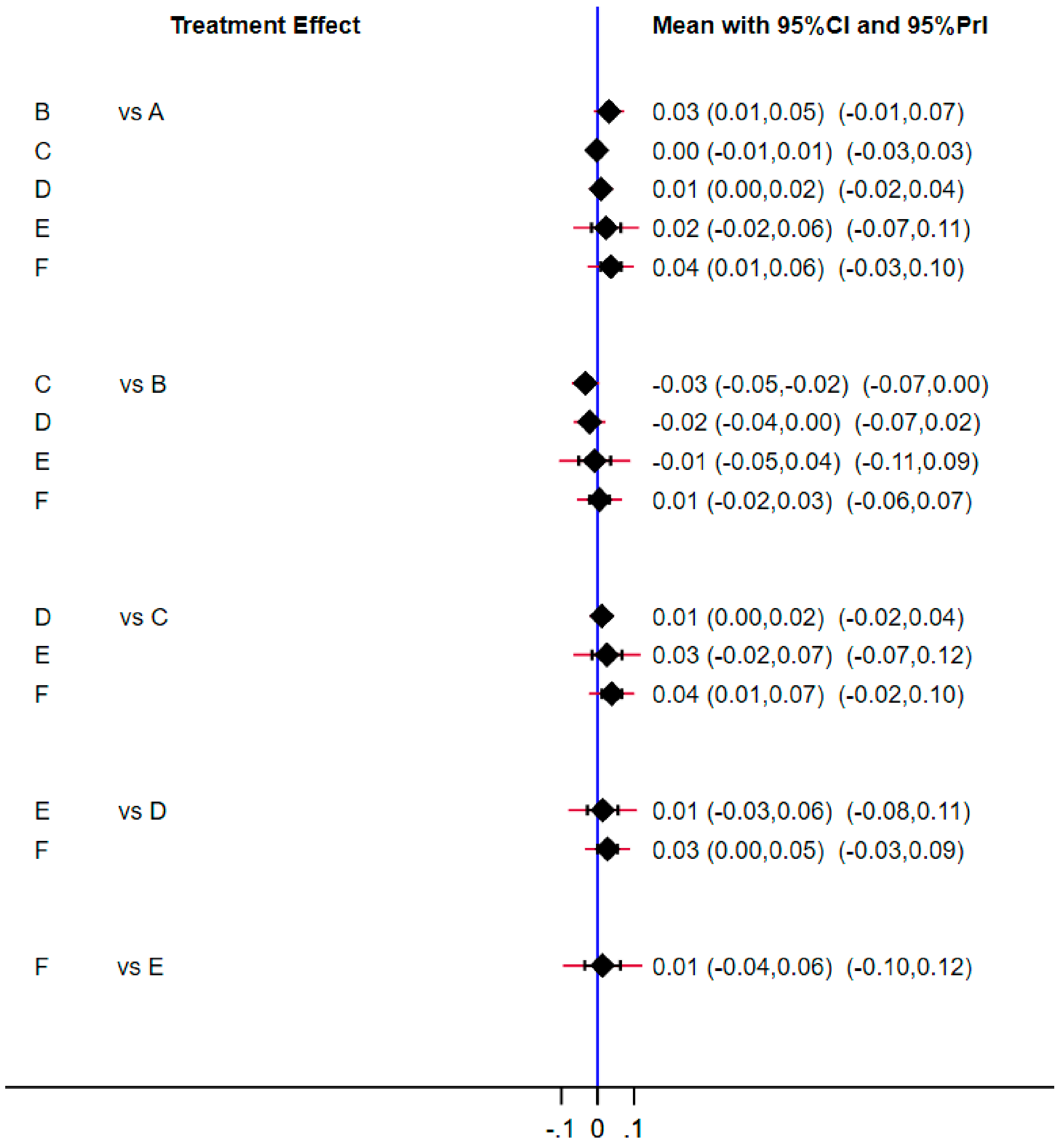

Figure S18. Effect size for TB BMD of milk-derived protein vs. soy protein and isoflavone using forest plots.

A: placebo/no treatment; B: soy protein; C: isoflavone; D: soy protein + isoflavone; E: milk basic protein; F: casein + whey protein.

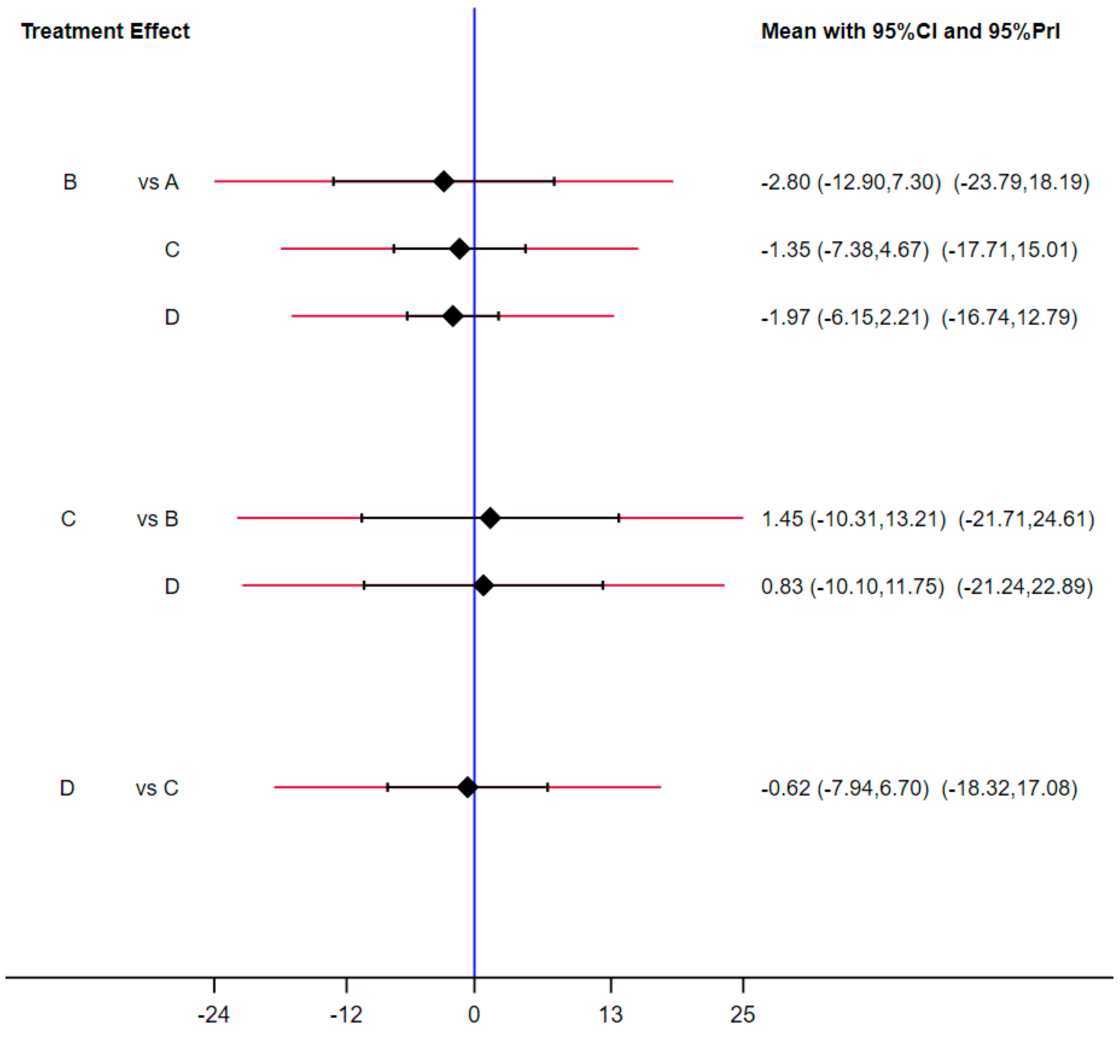

Figure S19. Effect size for OC of dairy products using forest plots.

A: placebo/no treatment; B: milk; C: milk + yogurt; D: milk powder.

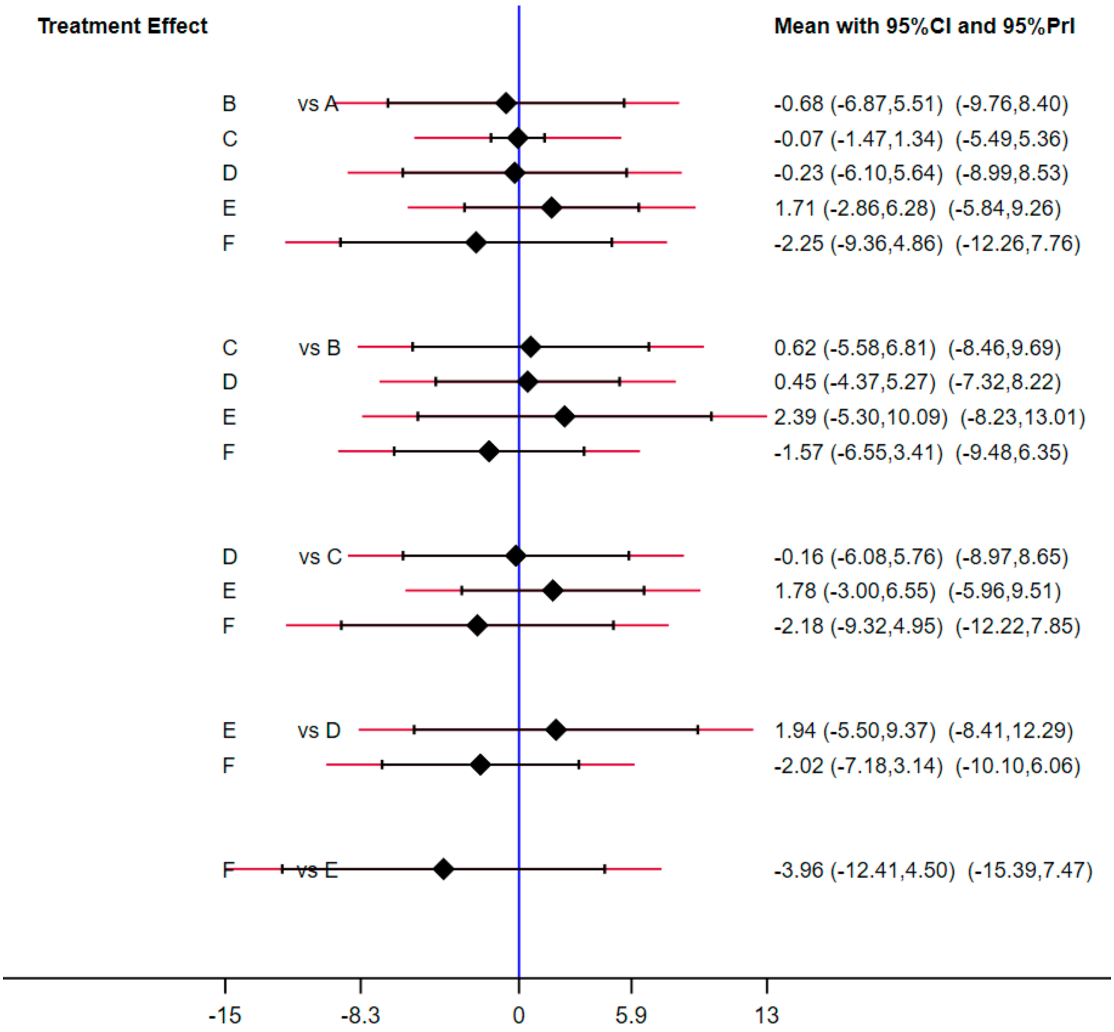

Figure S20. Effect size for OC of milk-derived protein vs. soy protein and isoflavone using forest plots.

A: placebo/no treatment; B: soy protein; C: isoflavone; D: soy protein + isoflavone; E: milk basic protein; F: casein + whey protein.

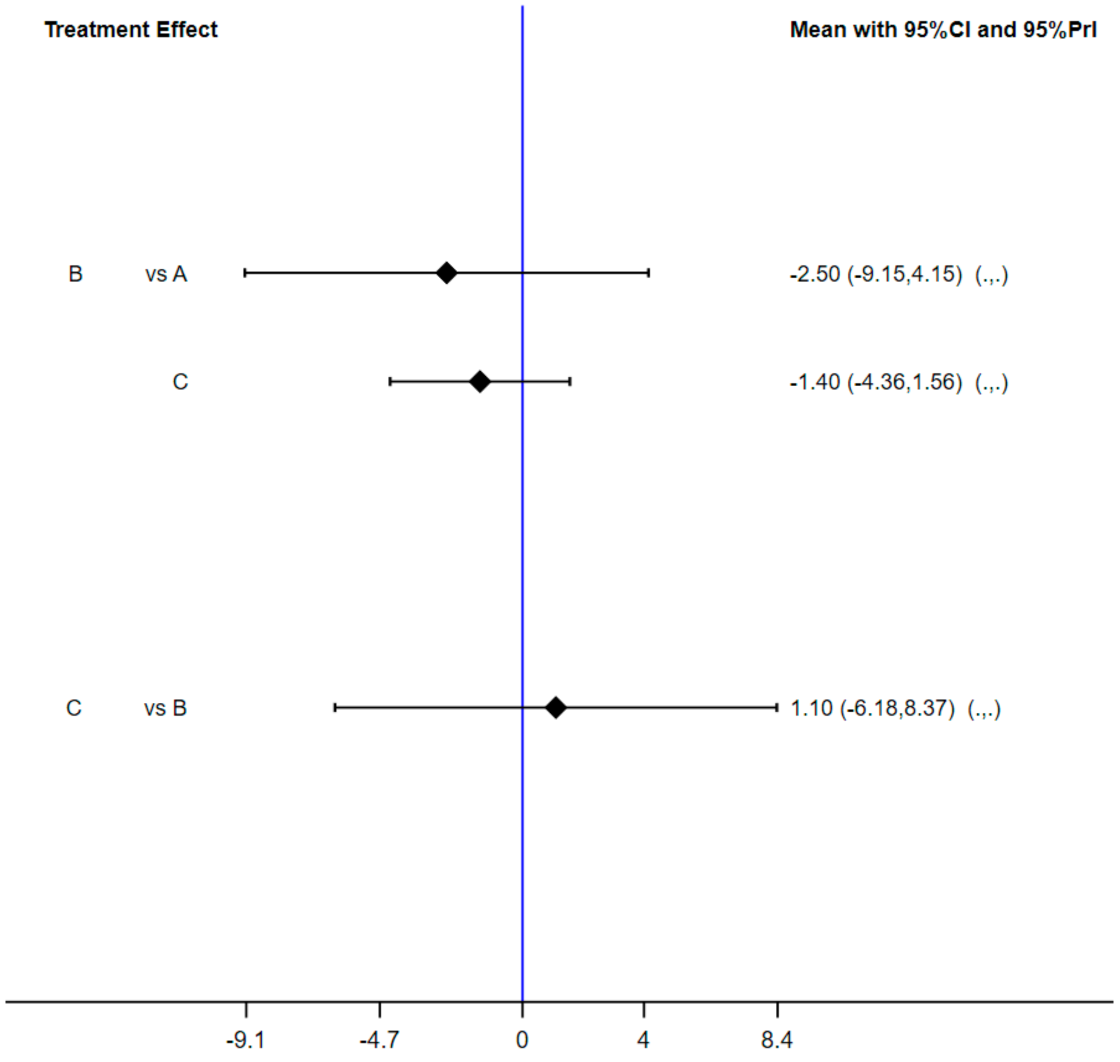

Figure S21. Effect size for DPD of dairy products using forest plots.

A: placebo/no treatment; B: milk; C: milk powder.

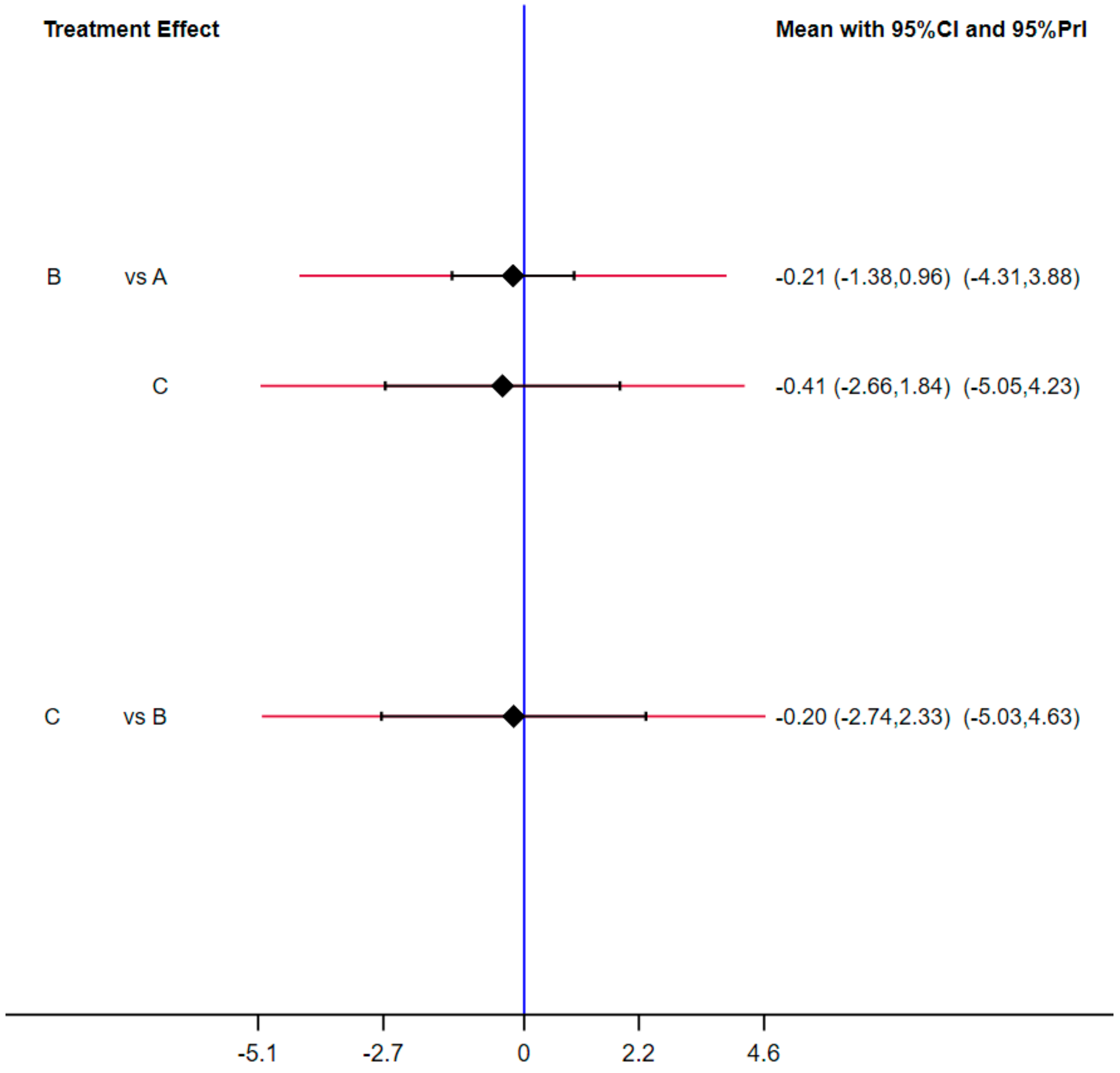

Figure S22. Effect size for DPD of soy protein vs. isoflavone using forest plots.

A: placebo/no treatment; B: isoflavone; C: soy protein + isoflavone.

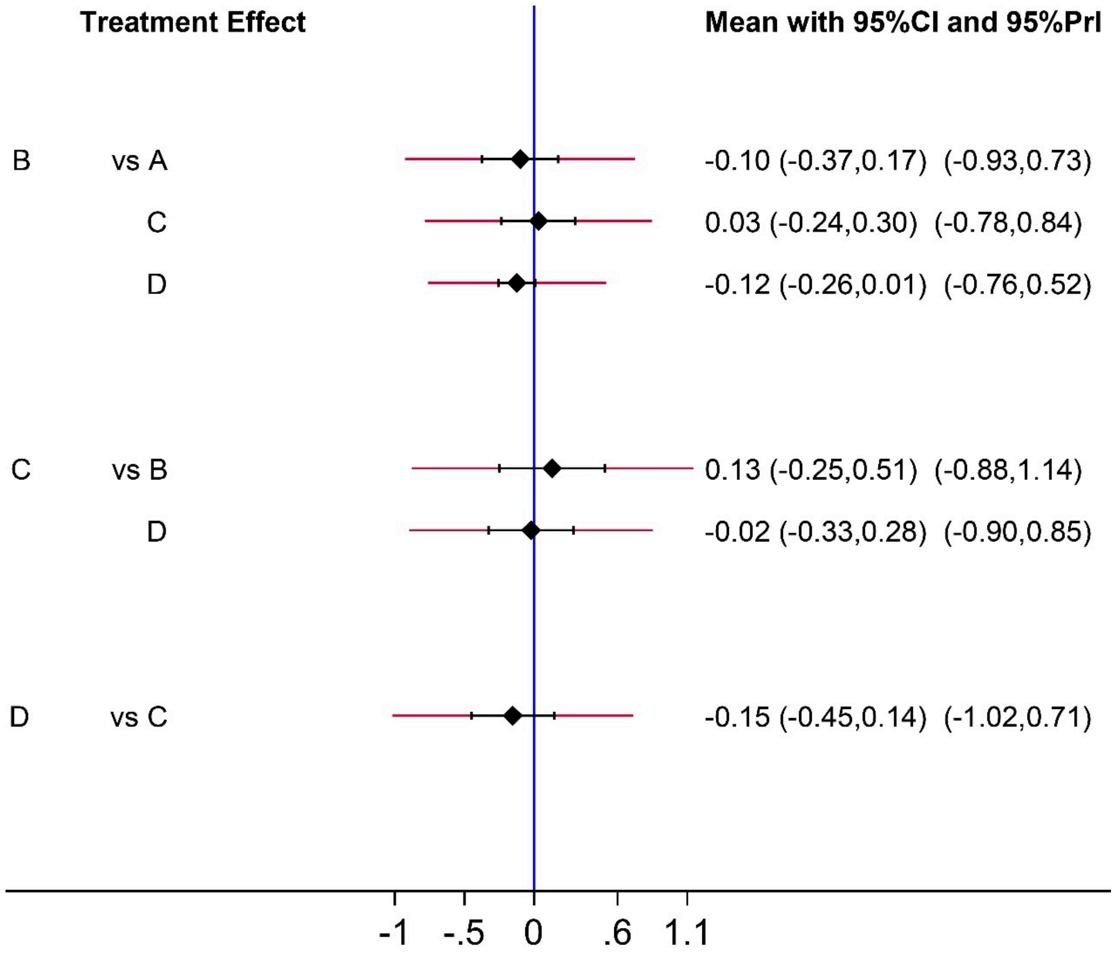

Figure S23. Effect size for CTx of dairy products using forest plots.

A: placebo/no treatment; B: milk; C: milk + yogurt; D: milk powder.

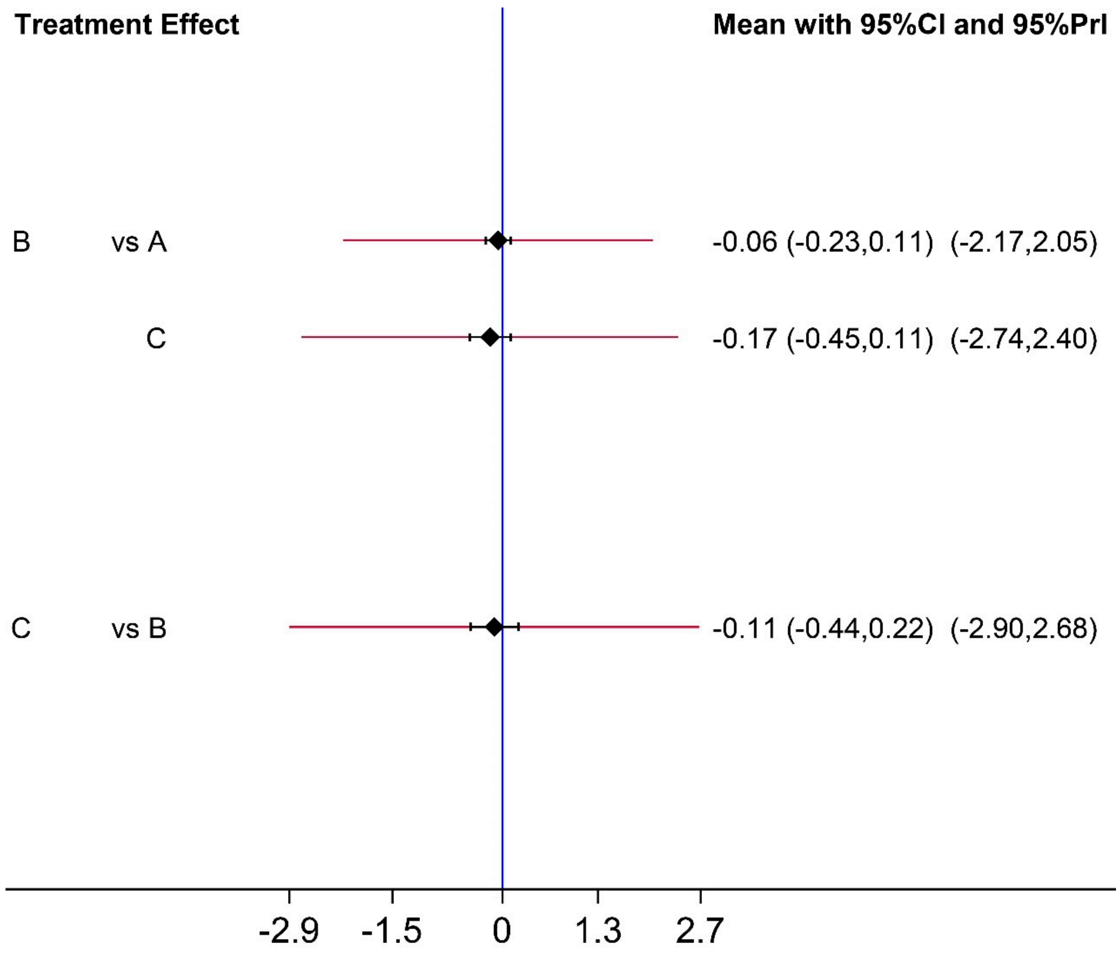

Figure S24. Effect size for CTx of milk-derived protein vs. soy isoflavone using forest plots.

A: placebo/no treatment; B: isoflavone; C: casein + whey protein.

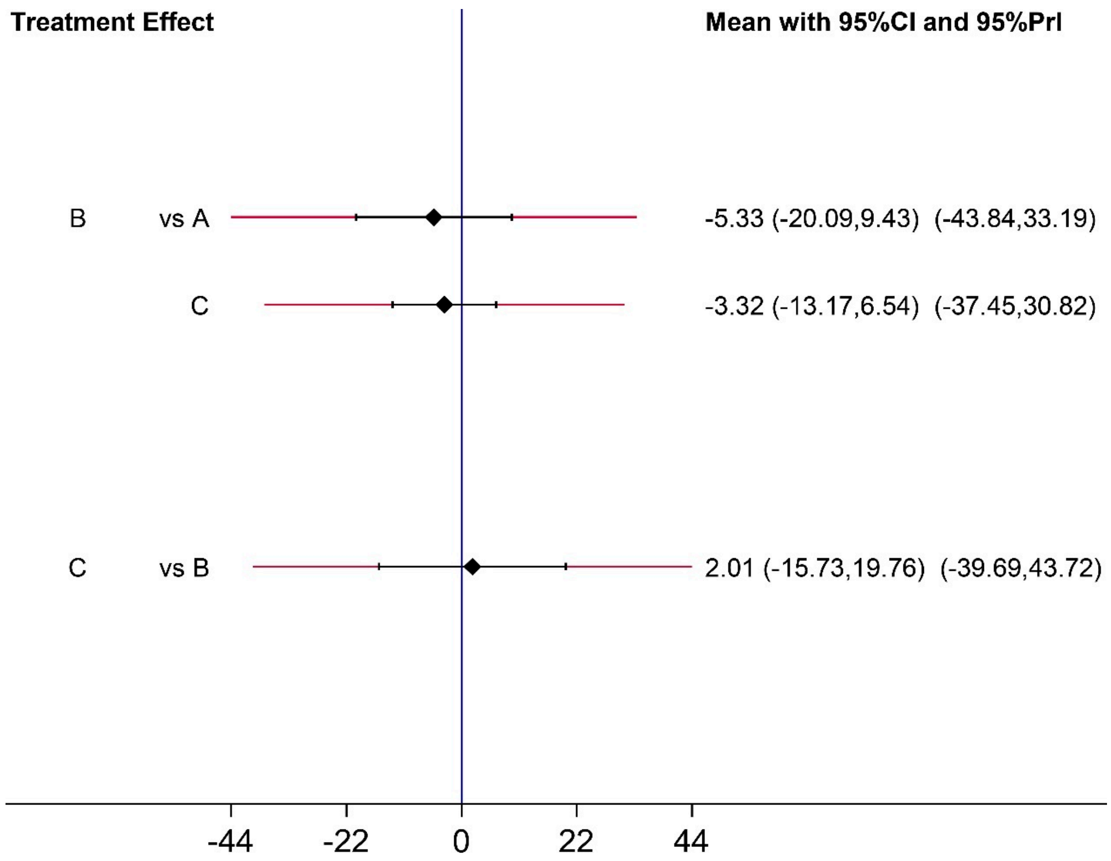

Figure S25. Effect size for PINP of dairy products using forest plots.

A: placebo/no treatment; B: milk; C: milk powder.

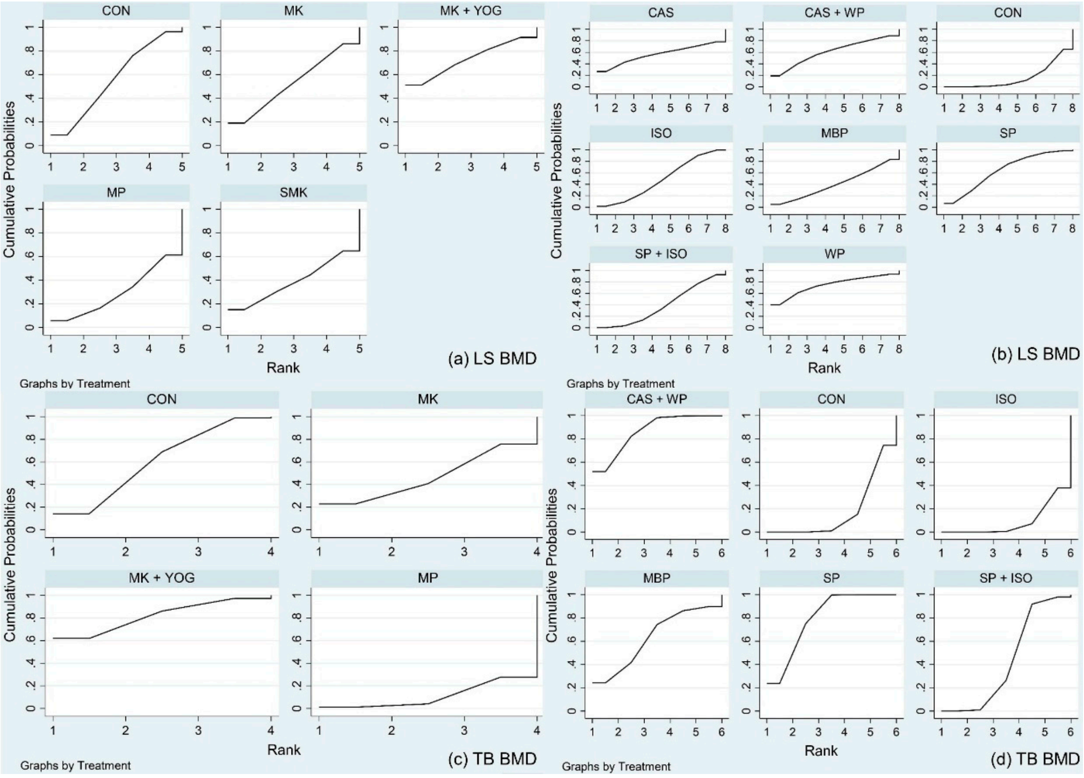

Figure S26. Ranking diagram of each intervention for lumbar spine (LS) BMD and total body (TB) BMD. CON: control (placebo/no treatment); MK: milk; MK + YOG: milk + yogurt; MP: milk powder; SMK: soymilk; SP: soy protein; ISO: isoflavone; SP + ISO: soy protein + isoflavone; MBP: milk basic protein; CAS: casein; WP: whey protein; CAS + WP: casein + whey protein.

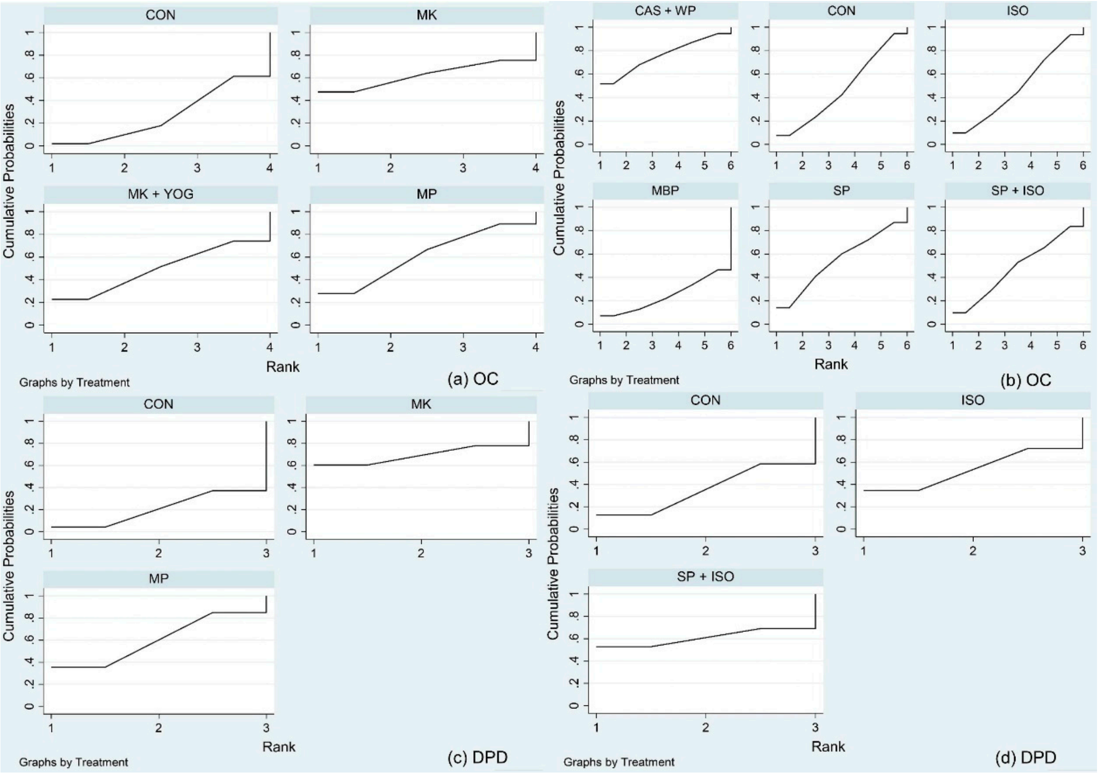

Figure S27. Ranking diagram of each intervention for osteocalcin (OC) and deoxypyridinoline and (DPD). CON: control (placebo/no treatment); MK: milk; MK + YOG: milk + yogurt; MP: milk powder; SP: soy protein; ISO: isoflavone; SP + ISO: soy protein + isoflavone; MBP: milk basic protein; CAS + WP: casein + whey protein.

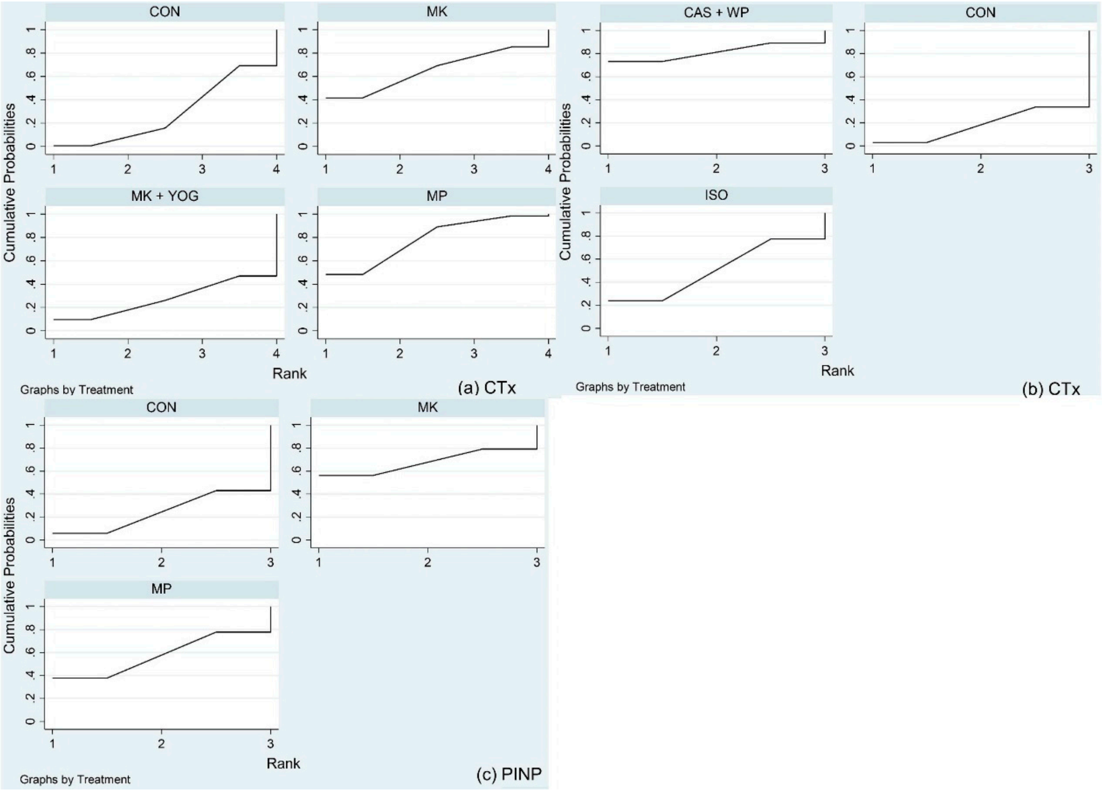

Figure S28. Ranking diagram of each intervention for C-terminal telopeptide of type I collagen (CTx) and procollagen type I N-terminal propeptide (PINP). CON: control (placebo/no treatment); MK: milk; MK + YOG: milk + yogurt; MP: milk powder ISO: isoflavone; CAS + WP: casein + whey protein.

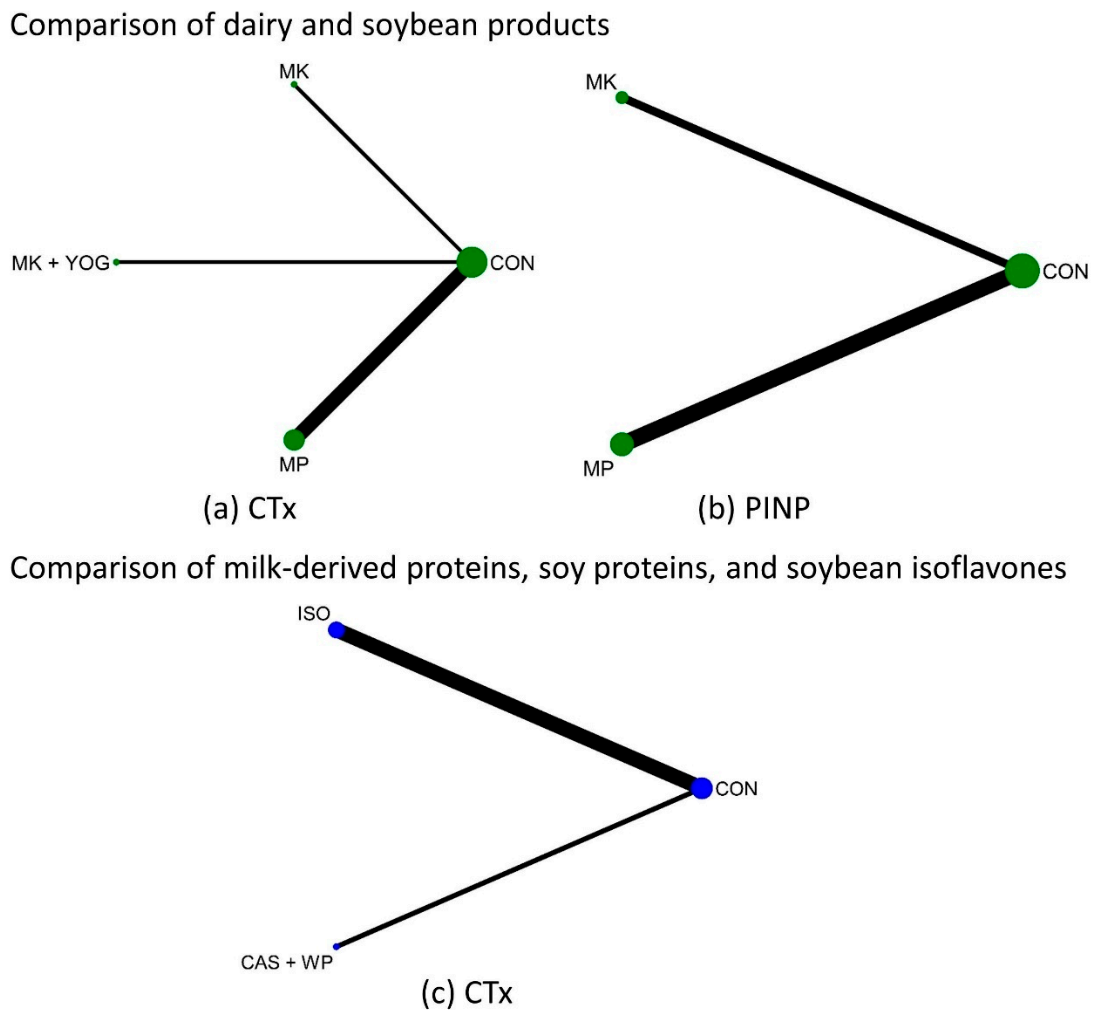

Figure S29. Network plots for C-terminal telopeptide of type I collagen (CTx) and procollagen type I N-terminal propeptide (PINP). Each node symbolizes an intervention, and each connecting line represents a direct comparison between two interventions. The node size and the line thickness reflect the number of participants evaluating each intervention and comparison, respectively. CON: control (placebo/no treatment); MK: milk; MK + YOG: milk + yogurt; MP: milk powder; ISO: isoflavone; CAS + WP: casein + whey protein.
